# The Redox Activity of Protein Disulphide Isomerase Functions in Non‐Homologous End‐Joining Repair to Prevent DNA Damage

**DOI:** 10.1111/acel.70079

**Published:** 2025-05-15

**Authors:** Sina Shadfar, Fabiha Farzana, Sayanthooran Saravanabavan, Ashley M. Rozario, Marta Vidal, Cyril Jones Jagaraj, Sonam Parakh, Esmeralda Paric, Kristy C. Yuan, Mariana Brocardo, Donna R. Whelan, Angela S. Laird, Julie D. Atkin

**Affiliations:** ^1^ Motor Neuron Disease Research Centre, Macquarie Medical School, Faculty of Medicine, Health and Human Sciences Macquarie University Sydney New South Wales Australia; ^2^ Holsworth Biomedical Research Centre, La Trobe Rural Health School La Trobe University Bendigo Victoria Australia

**Keywords:** ageing, cancer, DNA damage, DNA repair, double‐stranded DNA breaks, neurodegenerative conditions, zebrafish

## Abstract

DNA damage is a serious threat to cellular viability, and it is implicated as the major cause of normal ageing. Hence, targeting DNA damage therapeutically may counteract age‐related cellular dysfunction and disease, such as neurodegenerative conditions and cancer. Identifying novel DNA repair mechanisms therefore reveals new therapeutic interventions for multiple human diseases. In neurons, non‐homologous end‐joining (NHEJ) is the only mechanism available to repair double‐stranded DNA breaks (DSB), which is much more error prone than other DNA repair processes. However, there are no therapeutic interventions to enhance DNA repair in diseases affecting neurons. NHEJ is also a useful target for DNA repair‐based cancer therapies to selectively kill tumour cells. Protein disulphide isomerase (PDI) participates in many diseases, but its roles in these conditions remain poorly defined. PDI exhibits both chaperone and redox‐dependent oxidoreductase activity, and while primarily localised in the endoplasmic reticulum it has also been detected in other cellular locations. We describe here a novel role for PDI in DSB repair following at least two types of DNA damage. PDI functions in NHEJ, and following DNA damage, it relocates to the nucleus, where it co‐localises with critical DSB repair proteins at DNA damage foci. A redox‐inactive mutant of PDI lacking its two active site cysteine residues was not protective, however. Hence, the redox activity of PDI mediates DNA repair, highlighting these cysteines as targets for therapeutic intervention. The therapeutic potential of PDI was also confirmed by its protective activity in a whole organism against DNA damage induced in vivo in zebrafish. Hence, harnessing the redox function of PDI has potential as a novel therapeutic target against DSB DNA damage relevant to several human diseases.

## Graphical abstract

Schematic diagram illustrating the protective role of protein disulphide isomerase (PDI) against DNA damage via non‐homologous end‐joining (NHEJ), which repairs double stranded DNA breaks (DSBs). Induction of DNA damage results in the formation of γH2AX and p53‐binding protein 1 foci during NHEJ repair of DSBs. PDI (red circles) translocates into the nucleus following damage, where it co‐localises at DSB foci, facilitating NHEJ DNA repair. The PDI QUAD (green circles) mutant contains replacement of all four redox‐active cysteine residues with serine (SGHS SGHS) and it loses the redox activity but retains the chaperone function of PDI. The QUAD mutant is not protective against DNA damage, demonstrating that the redox activity of PDI is required for its function in NHEJ. Figure created with BioRender.com.

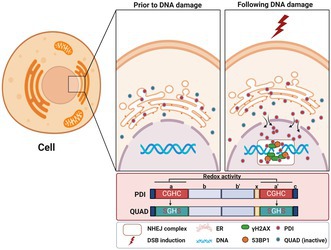

**Abbreviations**
53BP1: p53‐binding protein 1
APE1: apurinic/apyrimidinic endonuclease/redox factor‐1
BFP: blue fluorescent protein
DDR: DNA damage response
DMEM: Dulbecco's Modified Eagle's Medium high in glucose
DNA‐PK: DNA‐dependent protein kinase
DSBs: double‐stranded breaks
ER: endoplasmic reticulum
ERP57: ER protein 57
EV: empty vector
gH2aX: phosphorylated form of histone H2AX
H_2_O_2_
: hydrogen peroxide
HMGB1: high‐mobility group proteins 1
hpf: hours post‐fertilisation
HR: homologous recombination
Hsp: heat shock proteins
MMR: mismatch repair
NEB: New England Biolabs
Neuro‐2a: mouse neuroblastoma cells
NF‐κB: nuclear factor‐κB
NHEJ: non‐homologous end‐joining
NPM1: nucleophosmin
PBS: phosphate‐buffered saline
PDI: protein disulphide isomerase
RIPA: radioimmunoprecipitation assay buffer
ROS: reactive oxygen species
SD: standard deviation
SEM: standard error of the mean
siRNA: small interfering RNA
SR: super‐resolution microscopy
SSB: single‐stranded breaks
TBE: tris‐buffered saline
UI: un‐injected
UPR: unfolded protein response
UT: untransfected
WT: wildtype

## 1 Introduction

Ageing is a complex process (López‐Otín et al. 2023), and DNA damage is implicated as its major, unifying cause (Schumacher et al. 2021). Hence, targeting DNA damage and enhancing DNA repair may prevent age‐related dysfunction and disease. Cells are under constant attack from both exogenous and endogenous sources, and thousands of DNA lesions occur every day per cell (Curtin 2012). The DNA damage response (DDR) detects and repairs DNA damage to combat these threats. However, unrepaired DNA damage leads to apoptosis or senescence to prevent replicating the damaged genome. Hence, the DDR is essential for cellular health and viability. Disruption of DNA repair is well documented in diseases affecting neurons, including neurodegenerative conditions (Shadfar et al. 2023, 2022; Jagaraj et al. 2020), where ageing is the major risk factor (Jagaraj et al. 2024). However, there are no therapeutic strategies in clinical use to enhance DNA repair in these diseases. Targeting DNA repair pathways is also a therapeutic approach to kill rapidly dividing tumour cells due to their intrinsic genome instability compared to other cells. Identifying novel aspects of the DDR thus is of relevance to the pathophysiology of multiple diseases.

The most deleterious type of DNA damage is the formation of double‐stranded DNA breaks (DSBs), which are repaired by either homologous recombination (HR) or non‐homologous end‐joining (NHEJ). However, some cell types (such as post‐mitotic neurons) lack HR and thus rely entirely on NHEJ, which is much more error prone in comparison. Both p53‐binding protein 1 (53BP1) and DNA‐dependent protein kinase (DNA‐PK) perform pivotal functions in NHEJ (Lei et al. 2022). During DNA repair, DNA damage foci containing 53BP1 and phosphorylated histone H2AX (γH2AX) form at or near sites of DSBs and are a widely used marker of DNA lesions (Lei et al. 2022).

PDI is a highly abundant chaperone that possesses dual activities: catalysing the formation of disulphide bonds via oxidoreductase (redox) activity (Kersteen and Raines 2003) and refolding unfolded or misfolded proteins by chaperone activity (Kersteen and Raines 2003). The redox function of PDI is maintained by four cysteine residues located within two active sites in a “CXXC” motif (where “X” is any amino acid; Oka and Bulleid 2013). Up‐regulation of PDI is a cellular protective mechanism during the unfolded protein response (UPR) following endoplasmic reticulum (ER) stress, whereby misfolded/unfolded proteins accumulate in the ER. However, while PDI is abundant in the ER, it has now been detected in multiple other cellular locations (Lambert and Freedman 1985; VanderWaal et al. 2002), primarily the cytosol, mitochondria, cell surface, and extracellular matrix. However, a few reports have also detected PDI and other family members in the nucleus, where its functions remain unclear (Ali Khan and Mutus 2014). PDI is associated with cancer, cardiovascular diseases, and neurodegenerative conditions, but its roles in these diseases remain undefined.

In this study, we identify a novel function for PDI in the nucleus. PDI functions in NHEJ DNA repair of DSBs induced by two distinct mechanisms: oxidative stress and topoisomerase II inhibition. We demonstrated this in neuroblastoma cancer cell lines, and confirmed these findings in primary neurons. This protective function was mediated specifically by the redox, rather than chaperone, activity. PDI also co‐localised at DNA damage foci with γH2AX and 53BP1, and re‐localised to the nucleus after damage, implying that it has a direct role in DNA repair. PDI also inhibited oxidative DNA damage in zebrafish, confirming that it is protective in vivo. This is the first evidence showing that the redox activity of PDI functions in NHEJ, highlighting this as a potential therapeutic target for DNA repair mechanisms in human diseases.

## 2 Materials and Methods

### 2.1 Constructs

A pcDNA3.1(+) construct encoding PDI tagged with V5 was generously provided by Professor Neil Bulleid, University of Glasgow, UK (Jessop et al. 2009). A PDI ‘QUAD’ mutant, whereby all four active site cysteine residues were mutated to serine (SGHS SGHS), was as previously described (Parakh et al. 2020). A lentiviral construct expressing V5‐tagged PDI (pLV[Exp]‐ubiquitin C (UBC) > {hP4HB/V5/KDEL}) was obtained from VectorBuilder (vector ID: VB171221‐1156rmb). The PDI sequence was inserted into a mammalian gene expression lentiviral vector of size 8933 bp with an UBC promoter.

### 2.2 Cell Culture Maintenance

Neuro‐2a (mouse neuroblastoma) cells were purchased from Cellbank Australia (Lot: 10|004), and authenticated using short tandem repeat profiling by the European Collection of Authenticated Cell Cultures (ECACC: 89121404). NSC‐34 (motor neuron‐like) cells were generously provided by Professor Neil Cashman (University of British Colombia, Canada) and cultured in Dulbecco's Modified Eagle's Medium high in glucose (DMEM; Gibco), containing 10% (v/v) heat‐inactivated FCS at 37°C in a 5% CO_2_ humidified atmosphere. Cells were routinely examined for Mycoplasma contamination using a MycoStrip mycoplasma detection kit (InvivoGen, rep‐mys‐50).

### 2.3 Transfection

Neuro‐2a and NSC‐34 cells cultured on 13‐mm coverslips (Menzel‐Glasser) in DMEM (Gibco) in 24 well plates were transfected with 1 μg of V5‐tagged wild‐type PDI (PDI WT) or PDI QUAD (QUAD) constructs, or control empty vector (EV; pcDNA3.1(+)), using Lipofectamine 2000 (Invitrogen, 11668019) (2 μL per 1 μg of DNA) for 24 h following the manufacturer's instructions. Briefly, a day after seeding cells in 24 well plates, 50 μL of Opti‐MEM media was mixed with 1 μg of the required plasmid DNA and 51 μL of transfection mix (1:50) Lipofectamine‐2000 in Opti‐MEM, followed by a 20 min incubation. To prevent lipofectamine‐induced toxicity, the cell media was removed and replaced with fresh media 5 h following lipofectamine treatment.

### 2.4 Culturing and Transduction of Mouse Primary Cortical Neurons

Primary cortical neurons were cultured from C57BL/6 mouse embryos at embryonic day 16 (E16.5). The pregnant mice were purchased from Australian Bio Resources (ABR). Following dissection, cell dissociation, and counting as described previously (Fath et al. 2009), cells were frozen in 80% DMEM/10% FBS and 20% dimethylsulphoxide (DMSO; Sigma). To plate neurons from frozen stock, cryotubes were thawed in a 37°C water bath until the cell suspension was almost defrosted and then transferred to a sterile hood, where 1 mL of prewarmed DMEM/10% FBS was added drop‐wise. The cell suspension was then topped up to 10 mL in a 15 mL conical tube and centrifuged (300 *g*, 7 min at 21°C). The cell pellet was resuspended in 1 mL DMEM/10% FBS, and the viable cells were counted. Plating was performed as previously described (Fath et al. 2009). Briefly, the cells were plated onto poly‐d‐lysine‐coated glass coverslips (Gibco, A3890401) in DMEM/10% (vol/vol) FBS (plating medium; Invitrogen) and maintained in 2% B27‐supplemented Neurobasal medium (Gibco, 21103049) containing 0.25% GlutaMax (Gibco, 35050061). Neurons were transduced 6 days post‐plating with a lentiviral construct expressing V5‐tagged PDI (VectorBuilder, pLV[Exp]‐UBC > {hP4HB/V5/KDEL}; vector ID: VB171221‐1156rmb). Viral media was replaced 3 days post‐transduction. DNA damage was induced with 13.5 μM etoposide for 1 h. Immediately following DNA damage treatment, neurons were fixed with 4% paraformaldehyde (PFA). All cultures were maintained at 37°C in a 5% CO_2_ environment throughout the experiment.

### 2.5 Immunocytochemistry

Transfected and treated cells were washed with phosphate‐buffered saline (PBS; Invitrogen), fixed with 4% PFA and permeabilised in 0.1% Triton X‐100 in PBS for 10 min, followed by blocking in 1% BSA in PBS for 1 h. Cells were then incubated overnight at 4°C with the following primary antibodies (1:500): mouse anti‐V5 (Invitrogen, R960‐25), rabbit anti‐γH2AX (Novus Biologicals, NB‐100‐384), or rabbit anti‐53BP1 (Novus Biologicals, NB100‐304). The following secondary antibodies were added after three washes with PBS: anti‐rabbit Alexa Fluor 488 (Life Technologies, A11008); anti‐mouse Alexa Fluor 594 (1:500, Life Technologies, A21203); or anti‐rabbit Alexa Fluor 647 (1:500, Life Technologies, A21245). The samples were then incubated for 1 h at room temperature in the dark. The nuclei were counterstained with Hoechst 33342 (1:3000, Sigma‐Aldrich), and the cells were subsequently mounted using Faramount mounting medium (Dako, S3023) to preserve and enhance the immunoreactivity labelling before imaging.

### 2.6 Nissl Staining

Nissl stain was used as a neuronal marker to identify neurons in primary cultures. Primary cells were fixed in 4% PFA for 15 min at room temperature, followed by three washes with PBS. Following immunocytochemistry and Hoechst 33342 staining, neurons were then incubated with NeuroTrace 640/660 Deep‐Red Fluorescent Nissl Stain (Invitrogen: N21483) diluted in PBS (according to the manufacturer's instructions) for 20–30 min at room temperature in the dark. After staining, cells were washed three times with PBS and mounted with anti‐fade mounting medium (Faramount mounting medium [Dako, S3023]) for imaging.

### 2.7 Induction and Quantification of DNA Damage

DNA damage was induced by treatment with 13.5 μM etoposide for 30 min or 100 μM H_2_O_2_ for 1 h. Immunocytochemistry was performed as above and using confocal microscopy, the presence of γH2AX or 53BP1 foci was quantified as the number of foci per 100 cells. Cells containing large, overlapping foci could not be accurately counted, so were not included in the analysis.

### 2.8 Confocal Microscopy and Image Acquisition

Cells were photographed with 63×/na = 1.4 or 100×/na = 1.46 objectives on a Zeiss LSM 880 inverted confocal laser‐scanning microscope, equipped with an LSM‐TPMT camera (Zeiss). In multichannel imaging, photomultiplier sensitivities and offsets were set to a level at which bleed‐through effects from each channel were insignificant.

### 2.9 Single Molecule Super‐Resolution Imaging and Analysis

Following induction of DNA damage using etoposide (13.5 μM) for 30 min, cells were fixed and immunolabelled for anti‐P4HB (PDI; 1:2000, Abcam, ab3672); DNA‐PK (1:500, Abcam, ab18192); or 53BP1 antibodies (1:500, Novus Biologicals, NB100‐304) overnight, followed by secondary labelling with Alexa Fluor 532 (Invitrogen, 1:100) or Alexa Fluor 647 (Invitrogen, 1:100) for 1 h at 37°C. Cells were also stained with 10 μM DAPI for 10 min to label nuclei. Sample slides were mounted with a thin coverslip (#1.5). Imaging was performed using a home‐built single‐molecule super‐resolution (SR) microscope setup based around an Olympus IX83 inverted fluorescence microscope frame, 100 × 1.49 NA objective, and a photometrics Prime 95B sCmos camera. High laser excitation (532‐ and 640‐nm laser diodes at 3–5 kW/cm^2^) was used to induce single‐molecule photoswitching of labelled targets in PBS buffer containing 100 mM mercaptoethylamine and an oxygen scavenging enzyme cocktail (10% glucose, 400 μg/mL glucose oxidase and 40 μg/mL catalase). At least 10,000 frames of single‐molecule “blinking” events were captured at a high frame rate (8 ms exposure, 125 Hz) using Micromanager (Edelstein et al. 2010). Each channel was captured sequentially. Raw frames were processed in rapidSTORM 3.3 (Wolter et al. 2012) to localise emissions and render the super‐resolved image at 20 nm/pixel. Images were registered for chromatic aberration using bUnwarpJ (ImageJ plugin) after calibration using a coverslip coated with dispersed fluorescent beads (Tetraspecks).

For interaction analysis, the two‐coloured SR images were overlayed with the diffraction‐limited DAPI channel that indicated the nuclear boundary. With each nuclear area, interaction factor analysis (Bermudez‐Hernandez et al. 2017) was performed (random simulations = 10). An interaction factor value between 0 and 1 was assigned to each imaged nucleus, where a value closer to 1 indicated a non‐random association between the pair of targets (PDI and 53BP1, PDI and DNA‐PK). Interaction factor values that had corresponding *p*‐values > 0.2 were excluded from the dataset. Two‐sample t tests were performed using OriginPro.

### 2.10 Analysis of Apoptotic Nuclei

Following the induction of DNA damage and immunocytochemistry as above, cells undergoing apoptosis were identified by their nuclear morphology, as previously described (Parakh et al. 2020; Walker et al. 2010; Cummings and Schnellmann 2004), either condensed (≤ 5 μm in diameter) or fragmented (numerous condensed Hoechst‐positive structures in a single cell). Using ImageJ software (v. 1.53) (NIH), the percentage of cells with apoptotic nuclei was quantified from at least 100 cells expressing the desired plasmid.

### 2.11 Quantification of Endogenous PDI Expressed in the Nucleus

Following immunocytochemistry and induction of DNA damage as above, the proportion of endogenous PDI present in the nucleus relative to the whole cell was quantified using the “corrected total cell fluorescence (CTCF)” by ImageJ (Parakh et al. 2018). Briefly, 50 cells were selected using the freeform selection tool, and the fluorescence was quantified within the nucleus. An adjacent region without fluorescence was selected as background and the total fluorescence of the whole cell was calculated. The CTCF was then quantified using the formula; CTCF = Integrated Density − (Area of Selected Cell × Mean Fluorescence of Background Readings). CTCF for nuclear‐localised PDI was divided by CTCF for whole‐cell PDI to determine the relative proportion localised within the nucleus.

### 2.12 PDI Knockdown

To knockdown PDI, small interfering RNA (siRNA) was used following the manufacturer's protocol. Briefly, 100 nM PDI‐targeting siRNA (Dhrmacon On‐TARGETplus SMARTpool siRNA, DHA‐L‐HUMAN‐XX‐0010, horizon, USA) (CAGAUGAGCUGACGGCUGA; GAACAGACAGCUCCGAAGA; UAUCUGACUAUGACGGCAA; GAUAGCGACCACACUGAUA) or control scrambled siRNA (Dhrmacon On‐TARGETplus Non‐targeting pool; DHA‐D‐001810‐10‐05, horizon, USA) (UGGUUUACAUGUCGACUAA; UGGUUUACAUGUUGUGUGA; UGGUUUACAUGUUUUCUGU; UGGUUUACAUGUUUUCCUA) was resuspended in 1× Dharmacon siRNA buffer (horizon, B‐002000‐UB‐100, USA) in RNase‐free water. PDI siRNA or non‐targeting control siRNA were transfected into cells using Lipofectamine 2000 (Invitrogen, 11668019). To reduce toxicity induced by lipofectamine, the media was replaced and incubated for 48 h. Then, immunocytochemistry or western blotting was performed following induction of DNA damage as above.

### 2.13 Western Blotting

Cell lysates were prepared using radioimmunoprecipitation assay buffer (RIPA, 50 mm Tris–HCl, pH 7.5, 150 mm NaCl, 0.1% (w/v) SDS, 1% (w/v) sodium deoxycholate, and 1% (v/v) Triton X‐100) with 1% (v/v) protease inhibitor cocktail (Roche). Then, cells were centrifuged for 20 min at 13,000 rpm at 4°C, and the supernatant was stored at −80°C. The total protein in each sample was quantified using a Pierce BCA Protein Assay Kit (Thermoscientific), following the manufacturer's instructions. Soluble protein cellular lysates (25 μg) were analysed by 4%–15% gradient SDS‐PAGE gels and blotted onto nitrocellulose (Bio‐Rad). Membranes were blocked with 5% skim milk in 1 × PBS (Invitrogen) for 1.5 h and incubated with primary antibodies diluted in blocking buffer for 24 h at 4°C; anti‐γH2AX (1:1000, Novus Biologicals, NB‐100‐384); anti‐P4HB (PDI; 1:2000, Abcam, ab3672); anti‐rabbit Lamin B (1:3000, Abcam, ab16048); and GAPDH (1:4000, Proteintech, 60004‐Ig).

Twenty‐four hours post‐incubation, unbound primary antibodies were removed by washing in TBST (3 times × 5 min each) and the blots were incubated with secondary antibodies for 1 h at room temperature: goat anti‐mouse IgG or IgM HRP‐conjugated antibodies (1:4000, Merck EMD Millipore, Ap130P), or goat anti‐rabbit IgG peroxidase‐conjugated antibody (1:4000, Merck EMD Millipore, Ap132P). Immunoreactivity was revealed using the Clarity ECL Western Blotting Substrate kit (BioRad) and images were obtained using a BioRad ChemiDoc MP system, using Image Lab software (BioRad). The intensity of each band relative to GAPDH was quantified using ImageJ software (v. 1.47; National Institutes of Health).

### 2.14 Subcellular Fractionation

Neuro‐2a cells were transfected with either the V5‐tagged PDI construct or pcDNA3.1(+) EV. Twenty‐four hours post‐transfection, cells were treated with either 13.5 μM etoposide or DMSO for 30 min and washed with ice‐cold PBS. Then, cells were lysed with fractionation buffer (20 mM HEPES (pH 7.4), 10 mM KCI, 1.5 mM MgCl_2_, 1 mM EDTA). Following centrifugation (720 *g* for 5 min), the supernatant, containing the cytoplasm, membrane, and mitochondrial fractions, was transferred into fresh tubes. The pellet fraction, containing the nuclei, was washed with 500 μL fractionation buffer and centrifuged twice at 720 *g* for 10 min. Afterwards, the pellet was resuspended in 200 μL RIPA buffer with 0.1% SDS, followed by brief sonication (10 s on ice) to shear genomic DNA and homogenise the lysate. The primary supernatant was centrifuged at 10,000 *g* (8000 rpm) for 5 min. The resulting supernatant containing the cytoplasm was concentrated by centrifugation and frozen at −20°C until required.

The total amount of protein in each sample was quantified using a Pierce BCA Protein Assay Kit (Thermoscientific), according to the manufacturer's instructions. Then, 10–20 μg of protein samples were separated on 7.5% or 4%–15% (BioRad) SDS‐PAGE gels. Proteins were then transferred onto nitrocellulose membranes according to the manufacturer's instructions (BioRad) and blotted as above using polyclonal mouse anti‐P4HB (PDI) (1:2000, Abcam, ab3672), anti‐rabbit Lamin B (1:3000, Abcam, ab16048) or GAPDH (1:4000, Proteintech, 60004‐Ig) antibodies. After rinsing, the blots were incubated in peroxidase‐conjugated secondary antibodies (1:2000; Millipore) for 1 h at room temperature.

### 2.15 Single‐Cell Gel Electrophoresis Assay (Comet Assay)

A neutral Comet assay to specifically detect DSBs was adapted with some modifications (Olive and Banáth 2006). Briefly, Neuro‐2a cells transfected with either PDI or pcDNA3.1(+) EV for 24 h were treated with etoposide or vehicle (DMSO) for 30 min, trypsinised, and then suspended in ice‐cold PBS. The cells were mixed with 1% low‐melting agarose at 40°C and immediately transferred onto slides pre‐coated with 1% standard agarose. Coverslips were placed on top to create even thickness and cell distribution at 4°C to solidify. After removing the coverslips, the slides were immersed in lysis buffer (100 mM Na_2_EDTA, 10 mM Tris, 2.5 NaCl, pH 10.5, 1% Triton X‐100) for 1 h at 4°C before electrophoresis in neutral running buffer (90 mM Tris, 90 mM boric acid, 2 mM Na2EDTA, pH 8.5) at 20 V for 10 min. The dried slides were stained with 1X SYBR‐Gold (Molecular Probes; S11494). Comets were imaged using a Zeiss AxioImager epifluorescence microscope at 20× objective magnification. Comets that were clumped together or overlapped were excluded from the analysis because this disturbed migration and thus accurate quantification. Comet tail lengths were quantified using ImageJ as the distance from the centre of the nucleus to the tapered end of the tail. A minimum of 60 Comets from three biological replicates were scored.

### 2.16 Zebrafish Maintenance and mRNA Microinjection

All animal husbandry and experimental procedures were performed in compliance with the Animal Ethics Committee, Macquarie University (ARA 2015/034; ARA 2017/019; ARA 2017/03‐021) and the Internal Biosafety Committee, Macquarie University (NLRD 5201401007 and NLRD 5974 −52019597412350). Transgenic zebrafish expressing blue fluorescent protein (BFP) in their motor neurons on a TAB‐WT background—Tg(−7933mnx1: TagBFP) mq, were bred and maintained under standard zebrafish housing conditions. Adult zebrafish were mated, and the resulting embryos were collected for microinjection experiments. As previously described (Parakh et al. 2020), mKate2‐tagged PDI or QUAD was linearised by Not1 restriction enzyme digestion, purified using the QIAquick Gel Extraction Kit (Qiagen), and mRNA generated by in vitro transcription using a mMESSAGE mMACHINE SP6 transcription kit (Thermo Fisher Scientific). Then, mRNA encoding human PDI (WT or QUAD) fused to mKate2 (200 ng/μL) was injected via 0.905 nL droplets, into one to four cell stage zebrafish embryos using a Picospritzer II (Parker Instrumentation). At 24 h post‐fertilisation (hpf), embryos were screened for successful injection (expression of mKate2 red fluorescent protein) using an M165FC fluorescent stereomicroscope (Leica). Positive embryos (those expressing PDI or QUAD) were manually dechorionated (shells were removed) with forceps and raised in equal numbers with E3 medium (5 nM NaCl, 0.17 mM KCl, 0.33 mM CaCl_2_, and 0.33 mM MgSO_4_) in the dark at 28°C.

### 2.17 Western Blotting of Zebrafish Lysates

Protein lysates were prepared from zebrafish larvae (30 hpf) following removal of the protein‐rich yolk sac using deyolking buffer (55 mm NaCl, 1.8 mm KCl, 1.25 mm NaHCO_3_). The samples were then lysed in T‐PER tissue protein extraction reagent (Thermo Scientific) containing protease inhibitors (Complete ULTRA Tablets, Roche) using a dounce homogeniser. The total protein in each sample was quantified using a Pierce BCA Protein Assay Kit (Thermo Scientific) according to the manufacturer's instructions. Equal amounts of protein (20 μg) were loaded onto 7.5% or 4%–15% (BioRad) gels and transferred onto nitrocellulose membranes according to the manufacturer's instructions. Blots were pre‐incubated in blocking solution containing 5% (w/v) skim milk in Tris‐buffered saline (TBS), followed by 48 h incubation (γH2AX blots) or overnight (for PDI or GAPDH blots) at 4°C in primary antibodies diluted in blocking solution.

### 2.18 Statistics

Data are presented as mean value ± standard error of the mean (SEM) or standard deviation (SD), or median for the Comet assay. The statistical comparisons between group means were analysed using GraphPad Prism 10 software (Graph Pad software Inc.). For parametric data, a *t* test or one‐way ANOVA followed by a Tukey post hoc test was performed. For non‐parametric data, the Kruskal–Wallis test was used. The significance threshold was set at *p* = 0.05. The number of independent repeats (*n*), the statistical test used for comparison, and the statistical significance (*p* values) are specified for each figure panel in the representative figure legend.

## 3 Results

### 3.1 The Redox Activity of PDI Inhibits DNA Damage Induced by Etoposide in Neuronal Cells

**FIGURE 1:**
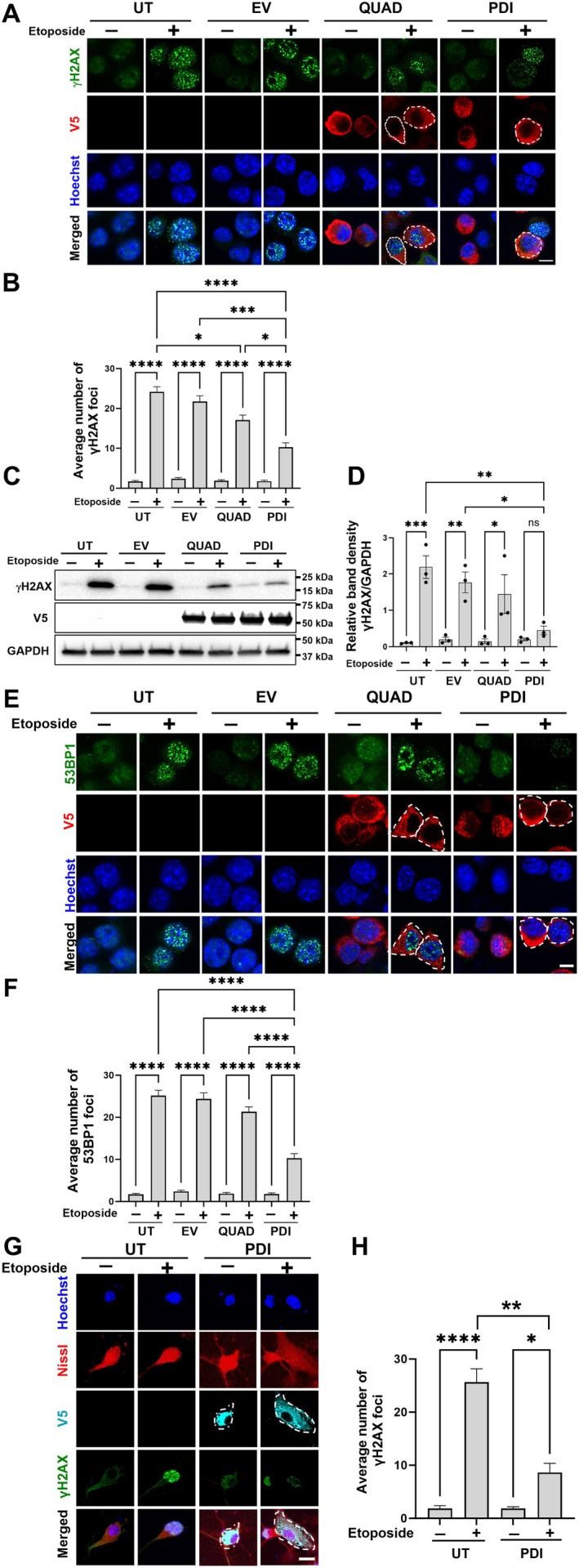
The redox activity of PDI is protective against DNA damage induced by etoposide. (A) Neuro‐2a cells were transfected with either pcDNA empty vector (EV), PDI, or QUAD tagged with V5, or untransfected cells (UT), and treated with 13.5 μM etoposide (or vehicle DMSO only) for 30 min at 24 h post‐transfection. Immunocytochemistry was performed using anti‐V5 (red) and anti‐γH2AX antibodies (green). Nuclei were stained with Hoechst (blue). Scale bar: 10 μm. (B) Quantification of the number of γH2AX foci in 100 cells in (A). Significantly less γH2AX foci were present in cells expressing PDI, but not QUAD, compared to untransfected and EV‐expressing cells. Kruskal–Wallis test, *n* = 3 independent replicates, mean ± SEM. *p < 0.05. ***p < 0.001, ****p < 0.0001. (C) Western blot analyses using anti‐γH2AX and anti‐PDI antibodies in Neuro‐2a cell lysates transfected with pcDNA EV, PDI tagged with V5, QUAD tagged with V5, or untransfected cells, following 30 min of etoposide treatment. (D) Quantification of relative band density of γH2AX in the blots in (C) using densitometry, GAPDH was used as a loading control. Significantly less γH2AX was present in cells expressing PDI, but not QUAD, following treatment with etoposide compared to UT or EV cells. One‐way ANOVA followed by Tukey's multiple comparison post hoc test, *n* = 3 independent replicates. All values represent mean ± SEM, *p < 0.05, **p < 0.01, ***p < 0.001, ns = non‐significant. (E) Immunofluorescence images of Neuro‐2a cells expressing PDI or QUAD tagged with V5 or pcDNA3.1 EV. Cells were fixed and immunostained with an anti‐53BP1 antibody (green) and V5 antibody (red) following 30 min of etoposide treatment. The number of 53BP1 foci was assessed in UT, pcDNA3.1 EV, PDI, and QUAD cells. Nuclei were stained with Hoechst (blue). Scale bar: 10 μm. (F) Quantification of the average number of foci per 100 cells shown in (A). Fewer 53BP1 foci were present in PDI cells compared to PDI QUAD cells, Kruskal–Wallis test, *n* = 3 independent replicates. ****p < 0.0001. (G) Mouse primary neurons were transduced with either PDI lentivirus tagged with V5 or untransfected cells (UT) and treated with 13.5 μM etoposide for 30 min at 24 h post‐transfection. Immunocytochemistry was performed using anti‐V5 (cobalt blue) and anti‐γH2AX antibodies (green). Nuclei were stained with Hoechst (blue) and deep‐red fluorescent Nissl stain was used for visualising neurons. Scale bar: 10 μm. (H) Quantification of the number of γH2AX foci in 30 neurons in (G). Significantly less γH2AX foci were present in neurons transduced with PDI compared to untransfected neurons. Kruskal–Wallis test, *n* = 3 independent replicates. All values represent mean ± SEM. *p < 0.05, **p < 0.01, ****p < 0.0001.

To investigate whether PDI is protective against DNA damage in vitro, Neuro‐2a, a mouse neuroblastoma cell line, was transfected with PDI tagged with V5 or pcDNA3.1(+) empty vector (EV). Etoposide, which inhibits DNA topoisomerase II, was used to induce DNA damage. Topoisomerase II regulates the topological state of DNA by inducing single‐ and double‐stranded breaks to resolve tangles and supercoils (Montecucco et al. 2015; Muslimović et al. 2009). At 24 h post‐transfection, cells were treated with 13.5 μM etoposide or DMSO (0.003% v/v) as a control. DNA damage was assessed by γH2AX, a widely used marker that primarily detects DSBs (Firsanov et al. 2011; Kinner et al. 2008; Mah et al. 2010) by the presence of discrete foci. Immunocytochemistry for γH2AX and V5, followed by quantification using confocal fluorescence microscopy, revealed that significantly more γH2AX foci formed in all etoposide‐treated groups compared to DMSO (12.3‐fold less), confirming the induction of DNA damage (Figure 1A,B; ****p < 0.0001). Significantly fewer γH2AX foci formed in etoposide‐treated cells expressing PDI compared to both untransfected (UT, ****p < 0.001) and EV‐transfected cells (***p < 0.001; Figure 1A,B; 2.3‐fold and 2.1‐fold, respectively). Hence, PDI is protective against the induction of DNA damage induced by etoposide.

Next, we examined whether the redox or chaperone activity of PDI is protective against DNA damage using a previously described “QUAD” mutant lacking all four redox‐active cysteine residues, which loses the redox activity but retains the chaperone function (Parakh et al. 2020; Whiteley et al. 1997; Hashimoto et al. 2008). This mutant contains two serine residues instead of the two redox‐active cysteines in each active site, producing two SXXS motifs instead of the two native CXXC sequences. Quantification revealed there were no significant differences in γH2AX foci formed in cells expressing PDI QUAD compared to EV cells, but significantly more foci were formed in PDI‐QUAD compared to WT PDI cells (Figure 1A,B; 1.6‐fold; *p < 0.05). Hence, the QUAD mutant is not protective against DNA damage, revealing that the redox function of PDI mediates its protective activity. This finding also confirms that non‐specific protein overexpression is not responsible for the observed protective function.

To confirm these findings, western blotting for γH2AX of Neuro‐2a cell lysates was performed. Quantification revealed that except for PDI‐expressing cells, significantly more γH2AX was present in all populations following etoposide compared to DMSO treatment, confirming the induction of DNA damage (Figure 1C,D). However, significantly less γH2AX was present in etoposide‐treated cells expressing PDI compared to EV and UT cells (3.9‐fold, *p < 0.05, 4.9‐fold, **p < 0.01, respectively). Hence, these data confirm that PDI is protective against DNA damage induced by etoposide. In contrast, there were no significant differences in γH2AX levels in etoposide‐treated cells expressing PDI QUAD compared to UT or EV cells, confirming that the redox activity mediates this function. Western blotting using anti‐V5 antibodies confirmed that similar levels of PDI were expressed in all WT‐ and QUAD‐transfected cells; thus, expression differences do not account for these findings (Figure 1C,D; *p* > 0.05). Hence, the redox, but not the chaperone, function of PDI is protective against DNA damage induced by etoposide in Neuro‐2a cells.

As phosphorylation of H2AX occurs primarily in response to DSBs (Firsanov et al. 2011; Kinner et al. 2008), these findings imply that PDI is protective against DSB formation. 53BP1 is specifically associated with DSB repair, particularly NHEJ (Gupta et al. 2014). When a DSB occurs, 53BP1 is recruited to sites of damage during NHEJ, where it processes and ligates the broken DNA ends, forming specific DNA damage foci, similar to γH2AX (Gupta et al. 2014). Hence, 53BP1 foci formation was next examined in cells treated as described above, following immunocytochemistry for 53BP1 and V5. Confocal fluorescent microscopy and quantification confirmed induction of DNA damage in all groups after etoposide treatment (Figure 1E,F; 14.8‐fold, ****p < 0.0001). Moreover, significantly fewer 53BP1 foci were present in etoposide‐treated cells expressing PDI compared to UT and EV controls (Figure 1E,F; 2.4‐fold and 2.3‐fold, respectively, ****p < 0.0001). Hence, PDI is protective against etoposide‐induced DSBs. In contrast, there were no significant differences in PDI‐QUAD‐expressing cells compared to both control groups, but significantly more 53BP1 foci were formed in these cells compared to WT PDI cells (Figure 1E,F; twofold, ****p < 0.0001). Hence, these findings imply that the redox function of PDI is protective against DSB formation.

To confirm these findings, similar experiments were performed in another neuronal cell line. NSC‐34 cells are a hybrid between spinal cord motor neurons and a mouse neuroblastoma cell line, N18TG2 (Cashman et al. 1992). They display the physiological and morphological properties of motor neurons, including generation of action potentials, expression of neurofilament triplet proteins, and acetylcholine synthesis, storage, and release (Cashman et al. 1992). NSC‐34 cells were transfected with either PDI tagged with V5 or pcDNA3.1(+) EV and treated for 30 min with either 13.5 μM etoposide or DMSO at 24 h post‐transfection (Figure S1). Immunocytochemistry for γH2AX and V5 followed by confocal fluorescence microscopy and quantification confirmed the induction of DNA damage in all etoposide‐treated groups (Figure S1A,B; ****p < 0.0001). Moreover, significantly fewer γH2AX foci were formed after etoposide treatment in PDI‐expressing cells, compared to untransfected and EV cells (Figure S1A; ****p < 0.0001). Hence, PDI is also protective against DNA damage in motor neuronal NSC‐34 cells as well as Neuro‐2a cells.

Similarly, 53BP1 foci formation was next examined in NSC‐34 cells expressing PDI. Significantly more 53BP1 foci in etoposide‐treated cells compared to DMSO controls confirmed induction of DNA damage in all groups (Figure S1C,D). As in Neuro‐2a cells, PDI‐expressing cells displayed significantly fewer 53BP1 foci than untransfected and EV cells, following etoposide treatment (Figure S1D; ****p < 0.0001). Thus, these results reveal that PDI inhibits DSB DNA damage in NSC‐34 cells as well as in Neuro2a cells.

While Neuro‐2a and NSC‐34 cells are neuronal cell lines, they undergo the cell cycle, unlike post‐mitotic neurons. Hence, to confirm that these findings are also applicable to neurons, we expressed PDI using lentivirus and transduced primary cortical neuron cultures obtained from mice at embryonic day 16 (Figure 1G,H). The cells were confirmed to be neurons by Nissl staining and by their characteristic morphology. Immunocytochemistry for γH2AX and V5, followed by quantification using confocal fluorescence microscopy, revealed that significantly more γH2AX foci formed in etoposide‐treated neurons compared to DMSO‐treated cells (13.4‐fold), confirming the induction of DNA damage (Figure 1G,H; ****p < 0.0001). Moreover, significantly fewer γH2AX foci (2.9‐fold less) formed in etoposide‐treated primary neurons transduced with PDI lentivirus, compared to untransfected (UT) cells (Figure 1G,H; **p < 0.01) following DNA damage. Hence, these results demonstrate that PDI is protective against the induction of DNA damage induced by etoposide in post‐mitotic cortical neurons.

### 3.2 The Redox Activity of PDI Inhibits DNA Damage Induced by H_2_O_2_ in Neuronal Cells

Reactive oxygen species (ROS) such as H_2_O_2_ are the main source of oxidative stress in living organisms. They are highly reactive towards DNA and can induce both DSBs (Cantoni et al. 1991; Driessens et al. 2009) and SSBs (Winterbourn 2008; Eiberger et al. 2008; Gillard et al. 2004), particularly in neurons (Wang and Michaelis 2010). Oxidative DNA damage is widely reported in neurodegenerative disorders (Winterbourn 2008) and cancer (Srinivas et al. 2019; Caliri et al. 2021). Hence, we next used H_2_O_2_ to induce oxidative DNA damage given its relevance to normal cellular function (Bordoni et al. 2019).

**FIGURE 2:**
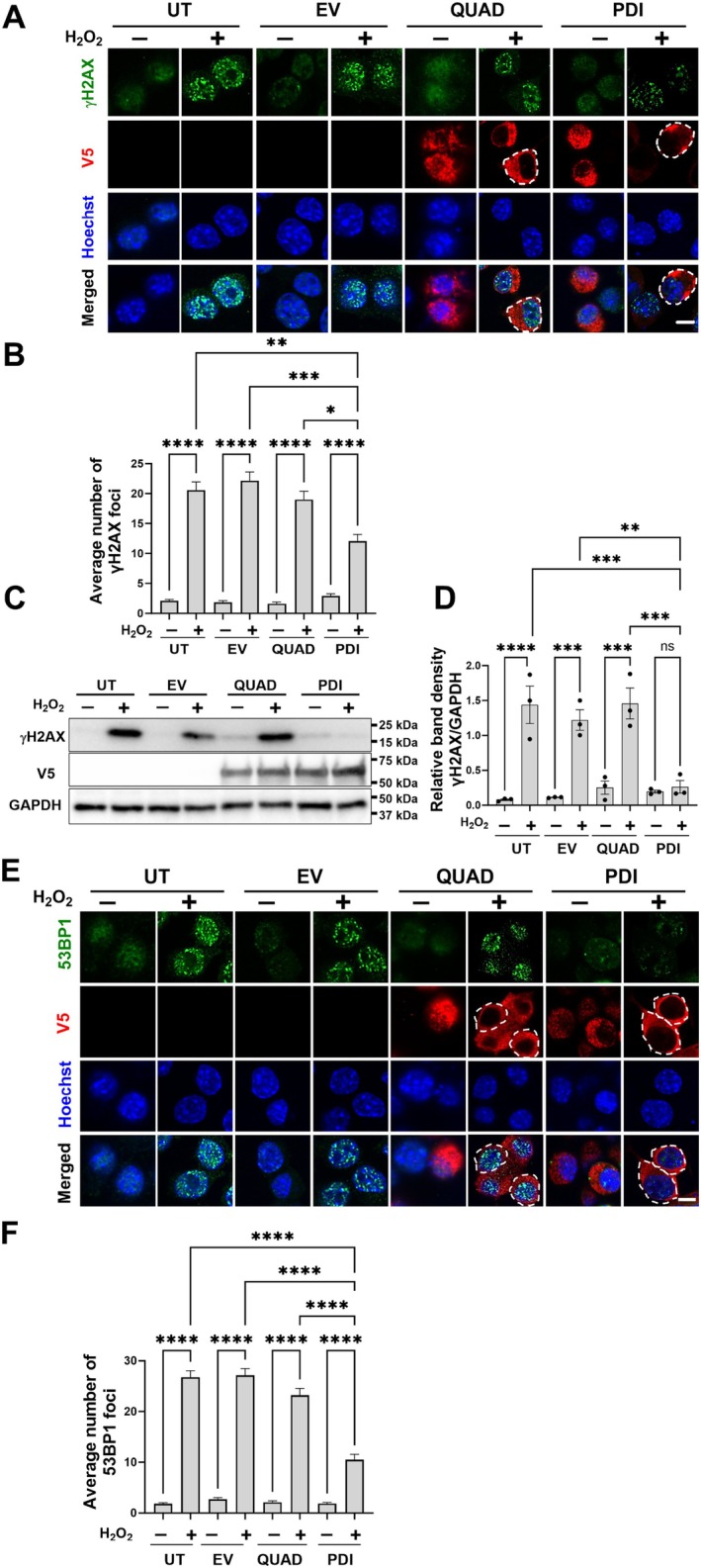
The redox activity of PDI prevents DNA damage induced by H_2_O_2_. (A) Neuro‐2a cells transfected with PDI tagged with V5, PDI QUAD tagged with V5, or pcDNA3.1 empty vector (EV), or untransfected cells (UT), were subjected to immunocytochemistry using anti‐γH2AX (green) and anti‐V5 antibodies (red), following 1h treatment with 100 μM H_2_O_2_ or vehicle (PBS). Nuclei were stained with Hoechst (blue). Scale bar: 10 μm. (B) Quantification of the average number of γH2AX foci per 100 cells. Significantly fewer γH2AX foci were present in PDI cells compared to PDI QUAD cells. Kruskal–Wallis test, *n* = 3 independent replicates, *p < 0.05, **p < 0.01; ***p < 0.001; ****p < 0.0001, mean ± SEM. (C) Western blotting using anti‐γH2AX and anti‐PDI antibodies of Neuro‐2a cell lysates transfected with pcDNA EV, PDI tagged with V5, PDI QUAD tagged with V5, or untransfected cells, following 1 h treatment with 100 μM H_2_O_2_ or PBS. (D) Quantification of the relative band density of γH2AX in blots shown in (C) using densitometry, GAPDH was used as a loading control. Significantly less γH2AX was present in cells expressing PDI following treatment with etoposide compared to UT or EV cells. One‐way ANOVA followed by Tukey's multiple comparison post hoc test, *n* = 3 independent replicates, mean ± SEM, **p < 0.01; ***p < 0.001; ****p < 0.0001, ns = non‐significant. (E) Neuro‐2a cells overexpressing PDI or PDI QUAD tagged with V5 (red) or EV were fixed and stained with anti‐53BP1 (green) and V5 antibodies (red), following 1h H_2_O_2_ treatment (100 μM) or PBS. Nuclei were stained with Hoechst (blue). Scale bar: 10 μm. (F) Quantification of the average number of foci per 100 cells. Significantly fewer 53BP1 foci were present in PDI cells compared to PDI QUAD cells following H_2_O_2_ treatment, Kruskal–Wallis test, 100 cells were counted in each experiment, *n* = 3 independent replicates. All values represent mean ± SEM, ****p < 0.0001.

Neuro‐2a cells were transfected with either V5‐tagged WT PDI, PDI‐QUAD, or pcDNA3.1(+) EV for 24 h and then treated with either 100 μM H_2_O_2_ or PBS for 1 h. Immunocytochemistry for V5 and γH2AX, then quantification following confocal fluorescence microscopy confirmed the induction of DNA damage in all etoposide‐treated compared to control groups (Figure 2A,B). Significantly fewer WT PDI‐expressing cells formed γH2AX foci compared to untransfected and EV‐transfected cells (Figure 2A,B; **p < 0.01, ***p < 0.001, respectively) following H_2_O_2_ treatment, revealing that PDI is protective against oxidative DNA damage. However, in PDI‐QUAD cells, no significant differences in γH2AX foci were present compared to UT and EV controls, and significantly more foci were evident than in PDI cells (Figure 2A,B; *p < 0.05). Hence, PDI QUAD is not protective against H_2_O_2_‐induced DNA damage, revealing that the redox function of PDI is protective against oxidative DNA damage.

Next, to confirm these findings, western blotting for γH2AX of Neuro‐2a cell lysates was performed. Quantification using densitometry revealed that except for PDI overexpressing cells, significantly more γH2AX was present in all populations following H_2_O_2_, compared to vehicle treatment, confirming the induction of DNA damage (Figure 2C,D). However, significantly less γH2AX was present in H_2_O_2_‐treated cells expressing WT PDI compared to EV and UT (4.6‐fold, **p < 0.01, 5.5‐fold, ***p < 0.01, respectively). Hence, PDI is protective against DNA damage induced by H_2_O_2_. In contrast, there were no significant differences in γH2AX levels in H_2_O_2_‐treated cells expressing QUAD compared to UT or EV cells, confirming that the redox activity of PDI is protective against oxidative DNA damage. Western blotting using anti‐V5 antibodies confirmed there were no significant differences in WT PDI and QUAD levels, thus differences in expression do not account for these findings (Figure 2C,D; *p* > 0.05). These data therefore confirm that unlike the chaperone activity, the redox function of PDI is protective against DNA damage induced by H_2_O_2_ in Neuro‐2a cells.

To further examine DSB repair, 53BP1 foci formation was next examined in H_2_O_2−_treated Neuro‐2a cells as above. Immunocytochemistry for V5 and 53BP1, followed by confocal fluorescent microscopy and quantification, confirmed induction of DNA damage (Figure 2E,F; ****p < 0.0001). Significantly fewer 53BP1 DNA damage foci were present after H_2_O_2_ treatment in PDI cells compared to untransfected and EV cells. Hence, PDI is protective against H_2_O_2_‐induced DNA damage (Figure 2E,F; ****p < 0.0001). In contrast, there were no differences in 53BP1 foci between PDI QUAD and UT or EV cells, but significantly more foci in PDI QUAD cells compared to WT PDI cells (Figure 2C,D; ****p < 0.0001). Hence, the redox activity of PDI is protective against DSB damage induced by H_2_O_2_.

### 3.3 The Protective Activity of PDI Against DNA Damage Involves NHEJ

**FIGURE 3:**
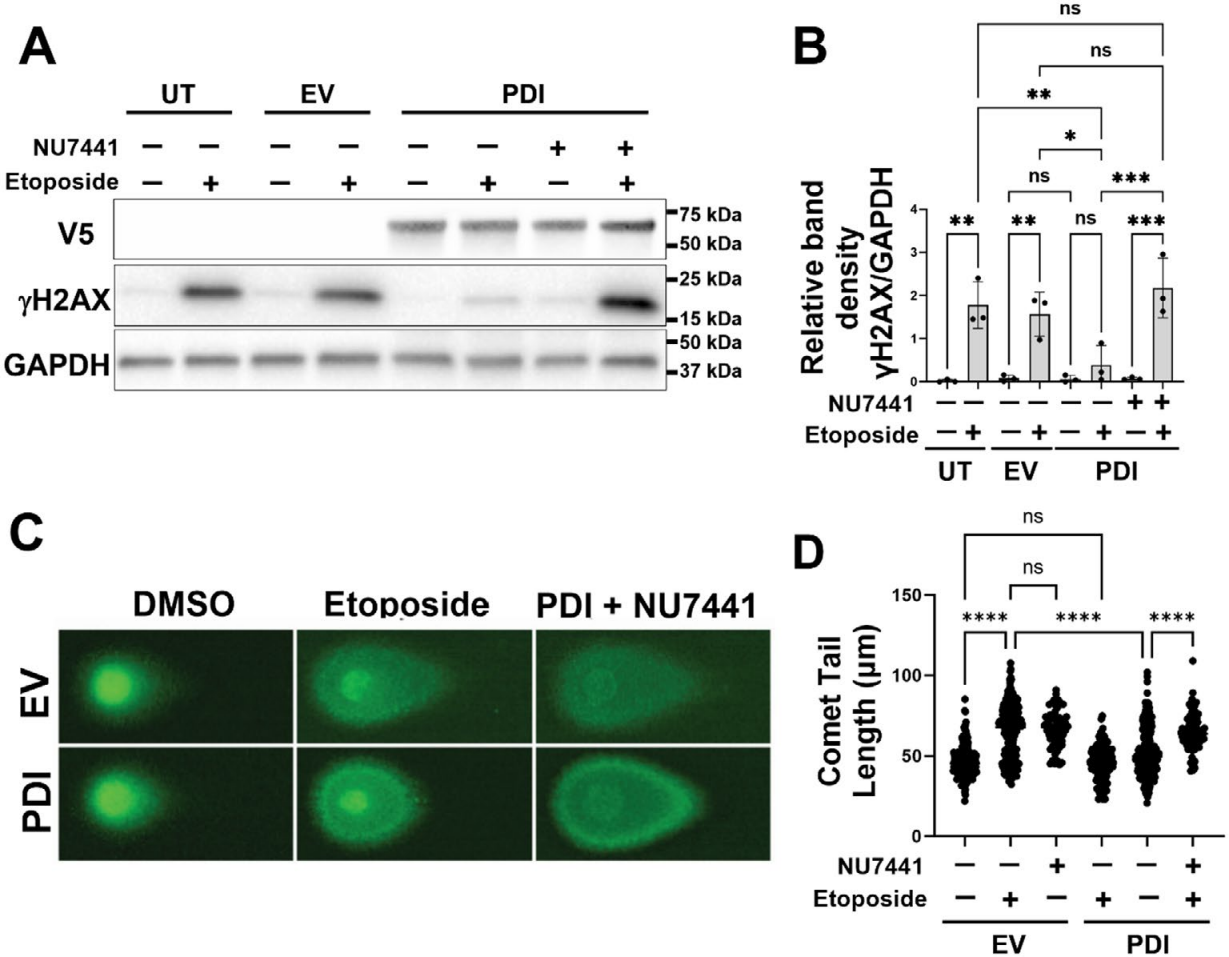
PDI is protective against DNA damage by NHEJ DNA repair. (A) Neuro‐2a cells were either untransfected (UT, first two columns) or transfected with either empty vector (EV) (second two columns), or PDI (last four columns). At 24 h post‐transfection, cells were treated with NU7441 for 1 h and 13.5 μM etoposide (or DMSO only) for 30 min. Then, cells were lysed and western blotting was performed using anti‐PDI and anti‐GAPDH antibodies. (B) Quantification of the blots in (A) using densitometry, GAPDH was used as a loading control. The graph depicts the relative band density of γH2AX to GAPDH compared to EV‐transfected cells. While γH2AX levels were significantly lower in PDI expressing cells following treatment with etoposide compared to UT or EV cells, they were significantly higher in PDI expressing cells following treatment with etoposide and NU7441 compared to PDI cells treated with etoposide only. One‐way ANOVA followed by Tukey's multiple comparison post hoc test, *n* = 3, *p < 0.05, **p < 0.01; ***p < 0.001, ns = non‐significant. (C) Representative images of neutral Comet assay of Neuro‐2a cells expressing either EV or PDI, treated with 13.5 μM etoposide for 30 min and NU7441 for 1 h. (D) Quantification of Comets expressed as the Olive Tail Moment (OTM) revealed less DNA DSBs in cells expressing PDI compared to EV‐expressing cells following etoposide treatment. More DNA DSBs were observed in cells expressing PDI following NU7441 treatment compared to PDI‐expressing cells, *n* = 3, ****p < 0.0001, ns = non‐significant.

As 53BP1 plays a crucial role in NHEJ (Driessens et al. 2009; Zhan et al. 2010), these results imply that the protective activity of PDI against DNA damage involves NHEJ. We examined this possibility using a selective inhibitor that impairs NHEJ: NU7441, which inhibits the catalytic subunit of DNA‐PK (Dong et al. 2018). Neuro‐2a cells were transfected with PDI or pcDNA3.1(+) EV. At 24 h post‐transfection, they were treated with NU7441 or vehicle control (DMSO) for 1 h and DNA damage was then induced using 13.5 μM etoposide for 30 min. Western blotting for γH2AX confirmed induction of DNA damage (Figure 3A,B). As above, in the absence of NU7441, DNA damage was significantly inhibited in cells expressing PDI compared to EV (*p < 0.05) and untransfected cells (**p < 0.01; Figure 3A,B). However, in the presence of NU7441, there were no significant differences in γH2AX levels in PDI expressing cells compared to EV and UT cells. Moreover, significantly more DNA damage was present in PDI cells treated with NU7441 compared to vehicle‐treated cells (Figure 3A,B; ***p < 0.001). Hence, impairment of NHEJ by NU7441 results in loss of the protective activity, implying that PDI prevents DNA damage by NHEJ repair.

To confirm the above results, we next performed a neutral Comet assay. The Comet assay is a direct and sensitive method to detect the presence of damaged DNA. Under neutral conditions, DSBs are specifically assessed. In EV cells treated with etoposide, there was a significant increase in the median Comet tail length compared to DMSO‐treated cells, confirming induction of DNA damage (Figure 3C,D; 1.5‐fold, ****p < 0.0001). In PDI‐expressing cells, a significantly shorter median tail length (1.4‐fold) was detected compared to EV cells, revealing that less damaged DNA accumulates in the presence of PDI (****p < 0.0001). However, in NU7441‐treated cells, the protective activity of PDI was lost: a significant increase in median tail length compared to PDI cells treated with vehicle (****p < 0.0001) was detected, with no significant difference compared to EV cells (Figure 3C,D). Hence, these results provide further evidence that PDI prevents DNA damage via NHEJ repair of DSBs.

### 3.4 PDI Knockdown Increases γH2AX Foci Following Etoposide and H_2_O_2_ Treatment in Neuro‐2a Cells

**FIGURE 4:**
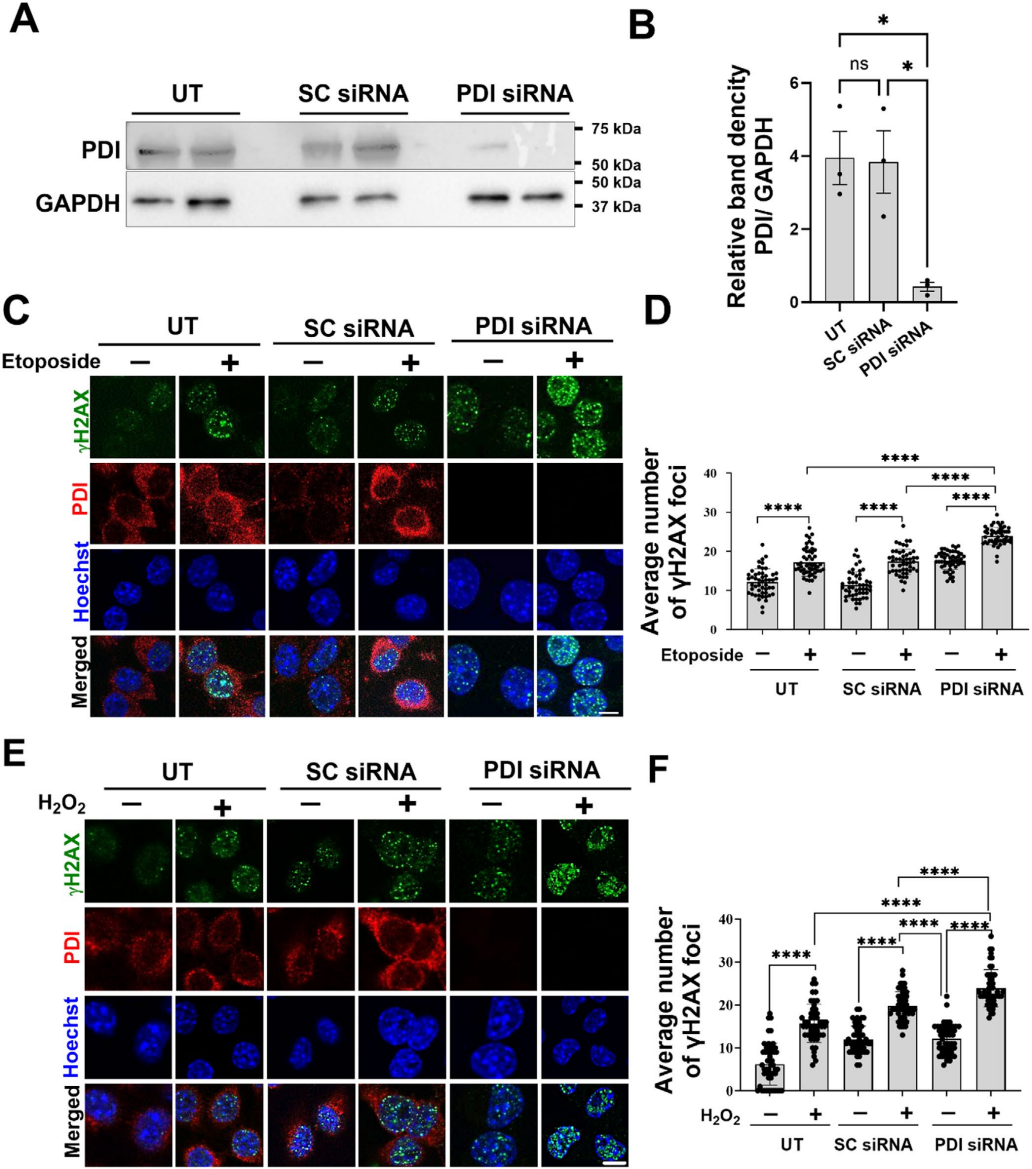
PDI knockdown increases DNA damage induced by etoposide and H_2_O_2_. (A) Neuro‐2a cells were either untransfected (UT, first two columns) or transfected with scrambled non‐targeting siRNA (SC siRNA) (second two columns), or PDI siRNA (third two columns). At 72 pours post‐transfection, cells were lysed and western blotting was performed using anti‐PDI and anti‐GAPDH antibodies. (B) Quantification of blots in (A) using densitometry, GAPDH was used as a loading control. The graph depicts the relative band density of PDI to GAPDH. Significantly less PDI was present in cells expressing PDI siRNA compared to UT‐ or SC siRNA‐cells. One‐way ANOVA followed by Tukey's multiple comparison post hoc test, *n* = 3, ****p < 0.0001, *p < 0.05, ns = non‐significant, mean ± SD. (C) Neuro‐2a cells were either untransfected (UT, first two columns), or transfected with scrambled siRNA (second two columns) or PDI siRNA (third two columns). At 72 h post‐transfection, cells were treated with 13.5 μM etoposide (or DMSO) for 30 min. Immunocytochemistry with γH2AX (green) and anti‐PDI (red) antibodies was performed and the nuclei were stained with Hoechst (blue). Scale bar represents 50 μm. (D) Quantification of the average number of γH2AX foci, indicating DNA damage, per 50 cells. Significantly more γH2AX foci were detected in Neuro‐2a cells transfected with PDI siRNA, compared to UT and scrambled siRNA‐transfected cells. Induction of DNA damage in cells treated with 13.5 μM etoposide compared to DMSO only treated cells for all groups was also confirmed by the presence of significantly more γH2AX foci. One‐way ANOVA followed by Tukey's multiple comparison post hoc test, 50 cells were counted from each group for *n* = 3 replicates. Values are presented as mean ± SD, ****p < 0.0001 (E) Neuro‐2a cells were transfected with either PDI siRNA (second two columns), or scrambled siRNA (third two columns). At 72‐h post‐transfection, these cells and untransfected (first two columns) controls were treated with 100 μM H_2_O_2_ (or PBS) for 1 h. Immunocytochemistry was performed using γH2AX (green) and anti‐PDI (red) antibodies and the nuclei were stained with Hoechst (blue). Scale bar represents 50 μm. (F) Quantification of the average number of γH2AX foci per 50 cells. A significant increase in γH2AX foci was detected in Neuro‐2a cells transfected with PDI siRNA compared to untransfected and scrambled siRNA‐transfected control cells. Significantly more γH2AX foci were detected in cells treated with 100 μM H_2_O_2_ compared to the PBS treated groups, confirming the induction of DNA damage. One‐way ANOVA followed by Tukey's multiple comparison post hoc test, 50 cells were counted from each group for *n* = 3 replicates. Values are presented as mean ± SD, ****p < 0.0001.

The above findings were obtained by over‐expressing PDI, which usually results in high, non‐physiological levels of protein expression. Hence, next a different paradigm was examined, by knocking down endogenous PDI using siRNA. Neuro‐2a cells were transfected with PDI‐targeting siRNA (100 nM) or the corresponding scrambled siRNA as a control for 72 h. Western blotting revealed that endogenous PDI was knocked down by 88% following siRNA treatment compared to scrambled siRNA‐treated and untransfected cells (Figure 4A,B; both *p < 0.05). At 72 h post‐transfection, cells were treated with 13.5 μM etoposide (or DMSO) for 30 min, and immunocytochemistry was performed for γH2AX and PDI (Figure 4C,D). Significantly more γH2AX foci were present in all etoposide‐treated cells compared to DMSO, indicating induction of DNA damage (Figure 4C,D; ****p < 0.0001). Moreover, significantly more γH2AX foci were present in PDI‐siRNA‐treated cells compared to scrambled siRNA and untransfected controls (Figure 4C,D; ****p < 0.0001). Hence, the knockdown of endogenous PDI increases DNA damage induced by etoposide in Neuro‐2a cells.

These data were confirmed using H_2_O_2_ to induce oxidative DNA damage. Neuro‐2a cells were transfected with PDI siRNA or scrambled siRNA as above and treated with 100 μM H_2_O_2_ for 1 h at 72 h post‐transfection. Immunocytochemistry for γH2AX and PDI (Figure 4E,F) and confocal fluorescent microscopy confirmed the induction of DNA damage following H_2_O_2_ treatment (Figure 4E,F; ****p < 0.0001). Significantly more γH2AX foci were detected in H_2_O_2−_treated cells with PDI knockdown compared to both untransfected and scrambled siRNA cells (Figure 4E,F; ****p < 0.0001). Thus, knockdown of endogenous PDI results in more DNA damage following H_2_O_2_ treatment, revealing that endogenous PDI normally protects against oxidative DNA damage.

### 3.5 PDI Relocates to the Nucleus Following Etoposide Treatment

**FIGURE 5:**
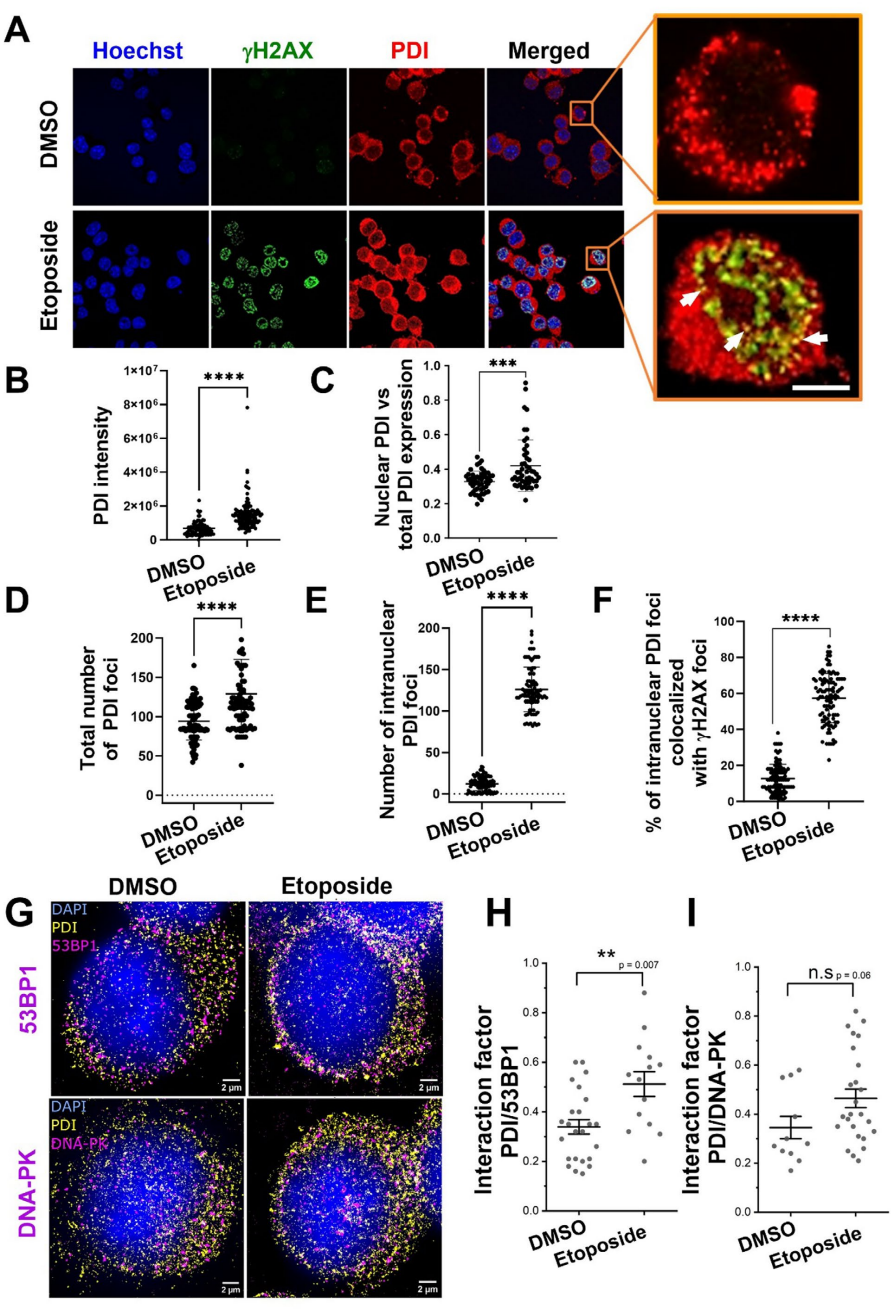
PDI is recruited to DNA damage foci in the nucleus following etoposide treatment. (A) Untransfected Neuro‐2a cells were treated with either DMSO (first column) or 13.5 μM etoposide for 30 min (second column). Immunocytochemistry was performed to examine PDI distribution and γH2AX foci following 30 min of etoposide or DMSO treatment in Neuro‐2a cells, using anti‐γH2AX (green) and anti‐PDI antibodies (red) and confocal imaging. Z‐stack confocal images were captured to quantify the co‐localisation between PDI and γH2AX foci. The arrows represent PDI and γH2AX co‐localisation (yellow). Nuclei were stained with Hoechst (blue). Scale bar represents 50 μm. Inset shows 3.5 times enlarged image of endogenous PDI intensity inside nucleus. (B) Quantification of the mean fluorescence of endogenous PDI in (A). A significant increase was observed in total PDI intensity following etoposide treatment, compared to the DMSO treated group, *n* = 3, unpaired *t* test, ****p < 0.0001. (C) Quantification of mean fluorescence of endogenous PDI in the nucleus (relative to total cellular PDI expression) in (A). A significant increase was observed in endogenous PDI intensity inside the nucleus following etoposide treatment, compared to the DMSO treated group. Mean fluorescence of nuclear PDI intensity compared to total cellular PDI for each group from *n* = 3 replicates. Unpaired *t* test with Welch's correction. Values are presented as mean ± SD, *n* ***p < 0.001 (D) Quantification of the total number of PDI foci following 30 min treatment with either DMSO or etoposide. Unpaired *t* test, ****p < 0.0001, *n* = 3 (E) Quantification of the average number of PDI foci within the nucleus per 100 cells. PDI foci were abundant in the nucleus of etoposide‐treated cells compared to DMSO‐treated cells, where very few were detected *n* = 3, unpaired *t* ****p < 0.0001. (F) Quantification of the percentage of intracellular PDI foci colocalised with γH2AX foci per 100 cells. Unpaired *t* test, mean ± SEM, *n* = 3, ****p < 0.0001 (G) Untransfected Neuro‐2a cells were treated with either DMSO (left) or 13.5 μM etoposide for 30 min (right). Immunocytochemistry was performed to examine PDI (yellow) and 53BP1 (magenta) distribution and colocalisation using single‐molecule super‐resolution microscopy. Nuclei were counter‐stained with 10 μM DAPI for 10 min (blue) and imaged using epifluorescence microscopy. Scale bar represents 2 μm. (H) Interaction factor quantification of the colocalisation of PDI and 53BP1 in the nucleus in (G). (I) Interaction factor quantification of the colocalisation of PDI and DNA‐PK in the nucleus in (G). For (E–H), *n* = 3, Single‐molecule super‐resolution imaging values are presented as mean ± SEM. Unpaired *t* test, ns = non‐significant; **p < 0.01. Following quantification, and for presentation purposes, the signal in the super‐resolution rendered images was dilated.

As DNA damage occurs predominantly in the nucleus, we next examined whether PDI is present in this compartment following etoposide treatment. Here, endogenous PDI was examined so that its normal physiological localisation following DNA damage could be examined. Untransfected Neuro‐2a cells were treated with 13.5 μM etoposide or DMSO for 30 min, followed by immunocytochemistry for γH2AX and PDI, and Hoechst staining to identify nuclei. Previous studies have shown that PDI can redistribute from a diffuse to a more punctate staining pattern, although this has been reported in the cytoplasm, not the nucleus (Yang et al. 2009; Bernardoni et al. 2013). In both DMSO‐ and etoposide‐treated cells, cytoplasmic foci were detected, consistent with previous studies (Figure 5A). However, discrete PDI foci were also detected within the nucleus of etoposide‐treated cells, but few were present in the nuclei of DMSO‐treated cells.

The total cellular expression of endogenous PDI (both cytoplasm and nucleus) increased significantly following induction of DNA damage by etoposide compared to DMSO, quantified by confocal microscopy and ImageJ (Figure 5A,B; ****p < 0.0001). The proportion of endogenous PDI within the nucleus relative to total cellular PDI was also quantified by confocal microscopy and ImageJ. Significantly more PDI localised in the nucleus following etoposide treatment compared to DMSO only (Figure 5A,C; ***p < 0.001), implying that following DNA damage, a proportion of cellular PDI translocates into the nucleus. The number of PDI foci per cell (nucleus or cytoplasm) also significantly increased (1.3‐fold increase) following treatment with etoposide (Figure 5A,D; ****p < 0.0001). Moreover, ImageJ analysis of the number of foci revealed that while only a few nuclear foci were present in DMSO‐treated cells, they were significantly more abundant in etoposide‐treated cells (Figure 5A,E; ****p < 0.0001). Hence, PDI forms foci in the nucleus following DNA damage.

These findings raise the possibility that PDI is recruited to DNA damage foci, where it could directly mediate repair functions at the DSB. In contrast, PDI may possess an indirect role in DNA repair, in which case it would be less likely to co‐localise with DDR proteins. This was next examined using confocal microscopy of γH2AX and PDI immunostained cells. Maximum projections of z‐stack images were created to quantify the co‐localisation between PDI and γH2AX foci using ImageJ. Significantly more PDI foci co‐localised with γH2AX (Figure 5A,F; ****p < 0.0001) following etoposide treatment (mean = 57.4%) compared to DMSO only (mean = 12.73%; 4.5‐fold increased; Figure 5A,F; ****p < 0.0001). Hence, together these data reveal that PDI is recruited to the nucleus following DNA damage, where it co‐localises with γH2AX foci.

We next investigated the recruitment of endogenous PDI to DNA damage foci (53BP1 and DNA‐PK) in more detail by super‐resolution microscopy (SR), using a previously described method to examine individual DSB sites (Konopka et al. 2020). Untransfected Neuro‐2a cells were treated with 13.5 μM etoposide or DMSO only for 30 min, followed by immunocytochemistry for γH2AX and PDI, and DAPI staining to identify nuclei. Single‐molecule localisation microscopy (Konopka et al. 2020) was performed to image the distribution of PDI with conventional dSTORM SR imaging of immunolabelled 53BP1 or DNA‐PK (Konopka et al. 2020). The resulting two‐colour images were examined using the “Interaction Factor” ImageJ plugin (Bermudez‐Hernandez et al. 2017) to evaluate the SR images of compact distributions in the nucleus (Konopka et al. 2020). In etoposide‐treated cells, PDI colocalisation with 53BP1 foci increased significantly, indicating recruitment and accumulation to DNA damage foci after DSB induction (Interaction Factor Mean [SEM] = 0.51 ± 0.05, Figure 5G,H). In contrast, DMSO‐treated cells displayed a lower Interaction Factor with 53BP1, indicative of low or more random colocalisation (Interaction Factor Mean [SEM] = 0.34 ± 0.03 Figure 5G,H). This confirmed that colocalisation artefacts due to crosstalk, bleed‐through, or photoconversion were largely absent, which were diminished using previously described techniques (Konopka et al. 2020). There was also a trend towards more colocalisation of PDI with DNA‐PK foci (Interaction Factor mean (SEM) = 0.46 ± 0.04, Figure 5G,I) compared to DMSO cells (Interaction Factor mean (SEM) = 0.35 ± 0.05 Figure 5G,I), but this did not reach statistical significance. The super‐resolved single‐molecule images represent the fluorophore localisations of endogenous PDI association with 53BP1 and DNA‐PK foci (Figure 5H). When rendered, equal weighting was given to each localisation, resulting in a snapshot of the spatial distribution of all labelled target proteins. Hence, presumably there was more variability in these results compared to the confocal images because only a small subset of each protein needs to respond to low levels of DNA damage. Quantifying endogenous proteins is therefore more representative of the normal physiological pathways but is also more prone to variability (Figure 5H,I). These results imply that PDI functions in DSB DNA repair within the nucleus, where it co‐localises with 53BP1.

We next examined whether PDI could also be detected in the nucleus following DNA damage in cells overexpressing PDI. Neuro‐2a cells were transfected with either V5‐tagged WT PDI or pcDNA3.1(+) EV for 24 h, then treated with either 13.5 μM etoposide or DMSO for 30 min. Immunocytochemistry for V5 and γH2AX following confocal fluorescence microscopy confirmed the induction of DNA damage in all etoposide‐treated compared to the control DMSO group. These images revealed the presence of PDI in the nucleus following DNA damage (Figure S2A). We examined this further by performing subcellular fractionation of cell lysates transfected with PDI as above to produce nuclear and cytoplasmic fractions. Western blotting using anti‐PDI, anti‐lamin‐B (as a nuclear marker), and anti‐GAPDH (as a cytoplasmic marker) antibodies was performed. The lack of GAPDH reactivity in the nuclear fraction confirmed that there was little contamination from the cytoplasm. Moreover, these analyses revealed that there was a significant increase in the levels of PDI in the nuclear fraction following etoposide treatment compared to DMSO‐treated cells (Figure S2B,D; **p < 0.01). In contrast, there was no statistically significant difference in the levels of PDI in the cytoplasm following etoposide treatment (Figure S2C,E; *p* > 0.05). Hence, these data confirm that both endogenous and over‐expressed PDI are recruited to the nucleus following DNA damage, where they co‐localise with γH2AX foci.

### 3.6 PDI Knockdown Enhances Apoptosis Following DNA Damage Induced by Etoposide and H_2_O_2_

**FIGURE 6:**
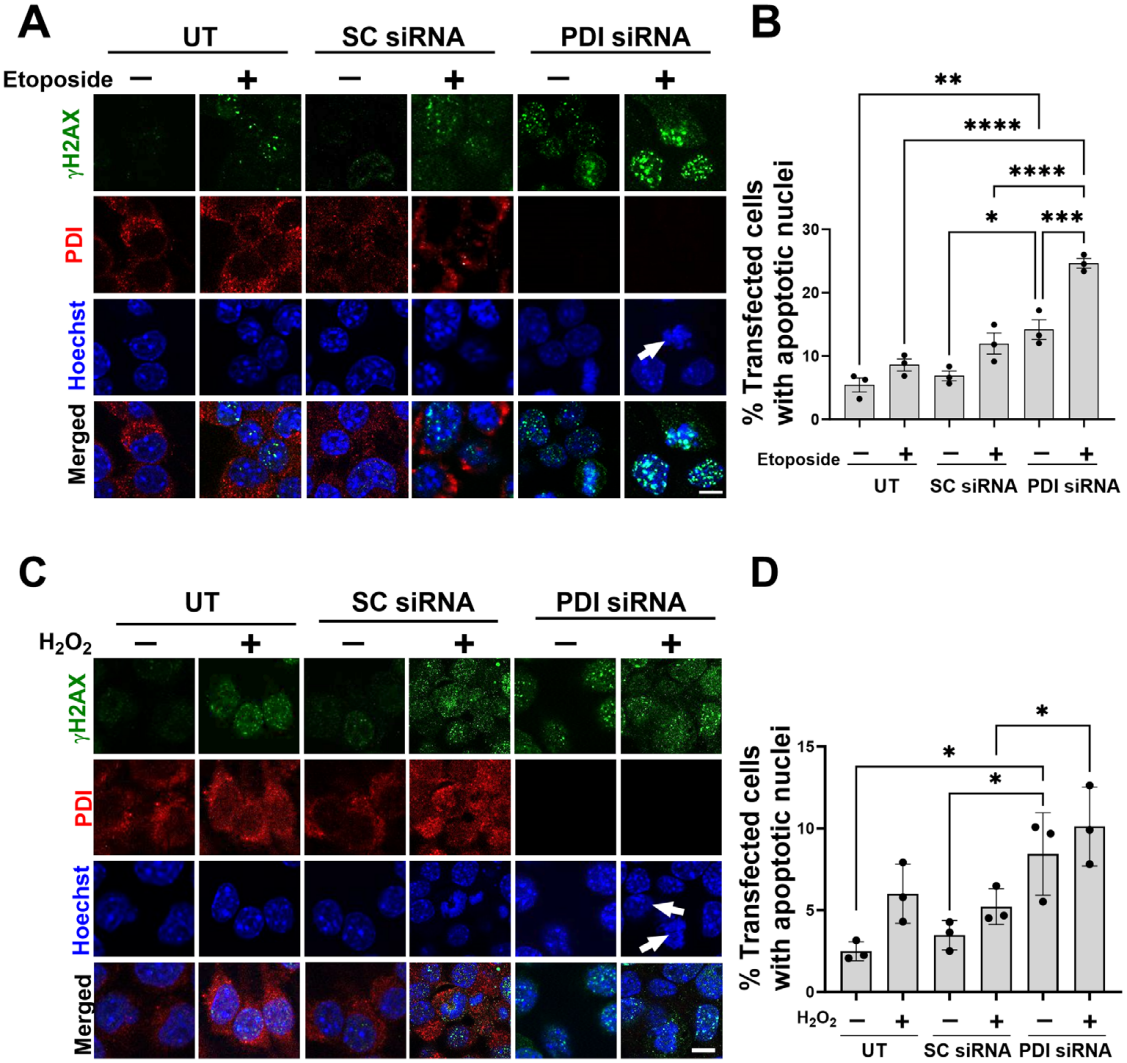
PDI is protective against apoptosis in Neuro‐2a cells following etoposide and H_2_O_2_ treatment. (A) Neuro‐2a cells transfected with either scrambled siRNA (SC, second two columns), PDI siRNA (third two columns), or untransfected cells (first two columns) were treated with 13.5 μm etoposide for 30 min at 72 h post‐transfection. Immunocytochemistry was performed using γH2AX (green) and anti‐PDI (red) antibodies, and the nuclei were stained with Hoechst (blue). Scale bar represents 50 μm. Arrows indicate Hoechst‐stained fragmented nuclei. (B) Quantification of apoptotic nuclei cells in Neuro‐2a cells in (A). A significant increase in apoptotic nuclei was observed between PDI siRNA‐transfected cells treated with etoposide compared to the DMSO treated group. Also, significantly more apoptotic nuclei in PDI siRNA cells compared to untransfected and SC siRNA transfected cells were detected, revealing that knockdown of PDI increases the formation of apoptotic nuclei in cells treated with either DMSO or etoposide. One‐way ANOVA followed by Tukey's multiple comparison post hoc test. Apoptotic nuclei were measured as a percentage of non‐apoptotic cells from 100 cells from each group for *n* = 3 replicates. Values are presented as mean ± SD, *p < 0.05; **p < 0.01; ***p < 0.001, ****p < 0.0001. (C) Neuro‐2a cells were transfected with either scrambled siRNA (second two columns), PDI siRNA (third two columns) or untransfected (first two columns) and treated with 100 μM H_2_O_2_ (or PBS) for 1 h after 72 h transfection. Immunocytochemistry was performed using γH2AX (green) and anti‐PDI (red) antibodies and the nuclei were stained with Hoechst (blue). Scale bar represents 50 μm. Arrows indicate Hoechst‐stained fragmented nuclei. (D) Quantification of cells in Neuro‐2a cells in (C) bearing apoptotic nuclei, treated with either PBS or 100 μM H_2_O_2_. Significantly more apoptotic nuclei in PDI siRNA‐transfected cells compared to scrambled siRNA‐transfected cells were detected, confirming that PDI plays a role in apoptosis following DNA damage by H_2_O_2_. One‐way ANOVA followed by Tukey's multiple comparison post hoc test. Apoptotic nuclei were measured as a percentage of non‐apoptotic cells from 100 cells from each group for *n* = 3 replicates. Values are presented as mean ± SD. *p < 0.05.

The DDR initially aims to repair DNA damage, but if prolonged or severe, apoptosis is induced. Hence, it was next examined whether endogenous PDI is protective against apoptosis in etoposide (or DMSO only) treated cells transfected with either siRNA targeting PDI or scrambled control above. DNA fragmentation occurs during apoptosis, and the presence of condensed or fragmented nuclei is an established method to monitor its induction (Parakh et al. 2020; Walker et al. 2010; Cummings and Schnellmann 2004). Apoptotic nuclei were thus identified by Hoechst in immunostained cells (γH2AX and PDI) with knockdown of PDI (Figure 6A). Few untransfected cells displayed fragmented nuclei with either DMSO (5.4%) or etoposide treatment (8.6%) (Figure 6B), revealing that under these conditions, etoposide induces DNA damage without significant apoptosis. Only 7% scrambled siRNA DMSO‐treated cells displayed fragmented nuclei, which increased slightly, but not significantly, to 12% following etoposide treatment, with no significant differences to untransfected cells (with or without DNA damage). In contrast, while only 14% PDI siRNA DMSO treated cells displayed fragmented nuclei, significantly more (25%, 2.3‐fold, ****p < 0.0001) were detected following etoposide treatment (Figure 6B). Importantly, significantly more apoptotic cells were present in etoposide‐treated PDI‐siRNA versus both scrambled‐siRNA‐transfected cells and untransfected cells (Figure 6A,B; **** *p* < 0.0001). Hence, endogenous PDI normally protects Neuro‐2a cells from apoptosis following DNA damage. There was also a significant increase in apoptotic nuclei in PDI siRNA cells compared to both untransfected (2.6‐fold, **p < 0.01) and scrambled‐siRNA cells in the absence of DNA damage (two‐fold, *p < 0.05; Figure 6B). Hence, knockdown of PDI also induces apoptosis by additional mechanisms other than DNA damage, consistent with previous observations (Muller et al. 2013).

This was further examined by 100 μM H_2_O_2_ treatment in Neuro‐2a cells treated above. Apoptotic nuclei were identified by Hoechst in immunostained cells (γH2AX and PDI) with knockdown of PDI (Figure 6C,D). Few untransfected cells displayed apoptotic nuclei in the presence (2.5%) or absence of H_2_O_2_ (6.0%) (Figure 6C,D) revealing that under these conditions, DNA damage is present without significant apoptosis. Similarly, only 3.5% scrambled siRNA cells displayed fragmented nuclei which increased slightly, but not significantly, following H_2_O_2_ treatment (5.2%) (Figure 6C,D). Moreover, significantly more cells with fragmented nuclei, indicating apoptosis, were detected in H_2_O_2_‐treated PDI siRNA cells (10.1%) compared to scrambled siRNA cells (**p < 0.01; Figure 6C,D). Hence, endogenous PDI normally protects Neuro‐2a cells from apoptosis following oxidative DNA damage. There was also a significant increase in apoptotic nuclei in PDI siRNA cells compared to both untransfected (2.6‐fold, **p < 0.01) and scrambled‐siRNA cells, in the absence of DNA damage (twofold, *p < 0.05; Figure 6B). These findings were consistent with the etoposide results, confirming that depletion of PDI induces apoptosis by mechanisms other than DNA damage. Hence, PDI protects Neuro‐2a cells from apoptosis following H_2_O_2_ treatment.

### 3.7 The Redox Activity of PDI Protects Against H_2_O_2_ ‐Induced DNA Damage In Vivo

Finally, the protective activity of PDI against DNA damage was examined in vivo to validate these in vitro findings. Zebrafish were selected as a model organism owing to the evolutionary conservation of key DDR proteins and DNA repair pathways (including NHEJ) with analogous functionality to humans (Cayuela et al. 2018). Furthermore, H_2_O_2_ can induce DSBs and NHEJ in zebrafish (Reinardy et al. 2013a) and thus was selected to induce DNA damage here.

The concentration of H_2_O_2_ required to produce DNA damage without significant toxicity was first established. Zebrafish larvae were incubated at 24 h post‐fertilisation (hpf) with increasing concentrations of H_2_O_2_ (500 μM, 1, 3, 5, 7, 10 mM) for 24 h in the dark (Figure S3). A dose‐dependent elevation in γH2AX expression was detected by Western blotting. Treatment at 3 mM increased γH2AX expression with less than 5% embryo mortality. Thus, this was selected as the optimised concentration to induce DNA damage (Figure S3).

**FIGURE 7:**
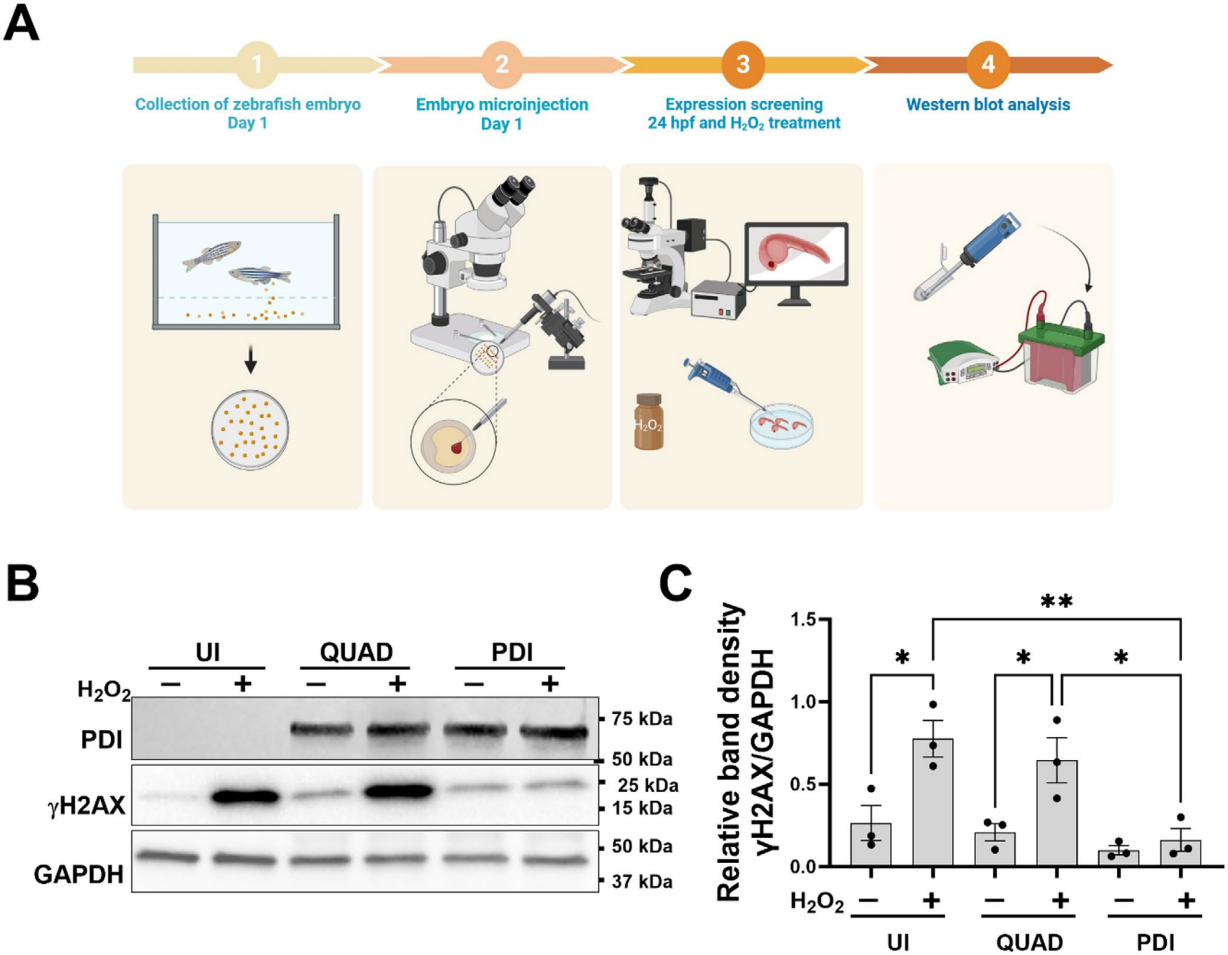
The redox activity of PDI is protective against the induction of DNA damage in vivo. (A) Schematic diagram illustrating the protocol used to induce and detect H_2_O_2_‐induced DNA damage following transient expression of PDI‐mKate2 and PDI QUAD‐mKate2 zebrafish embryos. A1: Zebrafish embryos were collected immediately after laying, then A2: they were Injected with PDI or QUAD mRNA at the 1–4 cell stage. A3: At 24 h post‐fertilisation (hpf), embryos were screened by fluorescence microscopy, and only those expressing mKate (red) were selected for further analysis. Following de‐chronisation, embryos were incubated with H_2_O_2_. A4: At 24 h post‐treatment, embryos were lysed and western blotting was performed. (B) Western blot analyses using anti‐γH2AX and anti‐PDI antibodies of zebrafish embryo lysates microinjected with PDI WT‐mKate2 or QUAD‐mKate2 following 24‐h treatment with 3 mM H_2_O_2_. UI, un‐injected. (C) Western blotting of lysates revealed that PDI, but not QUAD, was protective against H_2_O_2_‐induced DNA damage. One‐way ANOVA followed by Tukey's multiple comparison post hoc test, *p < 0.05, **p < 0.01, *n* = 3.

To examine the protective activity of PDI against DNA damage in zebrafish, mRNA encoding either PDI or QUAD tagged with mKate was microinjected into one to four cell stage zebrafish embryos, as previously reported (Parakh et al. 2020). At 24 hpf, embryos were treated with 3 mM H_2_O_2_ for 24 h (Figure 7A). Western blotting of un‐injected (UI) zebrafish embryo lysates for γH2AX confirmed that H_2_O_2_ treatment induced DNA damage (Figure 7B,C; *p > 0.05). Moreover, significantly less γH2AX was detected in H_2_O_2_‐treated embryos expressing PDI (Figure 7B,C; **p > 0.01) compared to UI lysates, revealing that PDI is protective against DNA damage induced by H_2_O_2_ in vivo. However, in H_2_O_2_‐treated PDI QUAD embryos, the levels of γH2AX were similar compared to the UI group, and significantly more DNA damage was present compared to H_2_O_2_‐treated PDI expressing embryos (Figure 7B,C, *p > 0.05). Hence, the redox activity of PDI is protective against H_2_O_2_‐induced DNA damage in zebrafish, confirming the in vitro findings and demonstrating that PDI is protective in vivo.

The results of this study and the paradigms examined are detailed in Table 1.

**TABLE 1:** Paradigms used to examine the protective role of PDI against DNA damage in this study.

Figure/s	PDI protection?	Model system	DNA damage inducer
Figures 1, and 4, 5, 6	Y	Neuro2a cells	Etoposide
Figure S1	Y	NSC‐34 cells	Etoposide
Figure 1	Y	Primary neurons	Etoposide
Figure 2	Y	Neuro2a cells	H_2_O_2_
Figure 7	Y	Zebrafish	H_2_O_2_

## 4 Discussion

Targeting DNA damage provides an opportunity to develop new therapeutic interventions for a range of diseases. Indeed, inducing DNA damage to selectively kill tumour cells is a widely used chemotherapeutic approach. Thus, understanding the mechanisms by which tumour cells can protect themselves against DNA damage is important to elucidate. However, the opposite strategy ‐ enhancing DNA repair to preserve cellular viability ‐ is not yet available therapeutically for diseases involving DNA damage‐induced cell death, such as neurodegenerative conditions. While PDI enhances cell survival (Parakh and Atkin 2015; Graven et al. 2002; Sullivan et al. 2003) and is associated with multiple human diseases, the underlying mechanisms remain poorly characterised.

In this study, we describe a new feature of the DDR. We show that PDI functions in NHEJ repair, protecting against DSBs, the most deleterious type of DNA damage. Many forms of chemotherapy induce DSBs, including the topoisomerase inhibitor used in this study, etoposide, and platinum‐based drugs such as cisplatin. Hence, identifying mechanisms to inhibit NHEJ in cancer cells is important in developing novel therapeutics. Importantly, NHEJ is the only DSB DNA repair mechanism available to neurons, implying that PDI prevents DNA damage induced during neurodegeneration. These findings are consistent with other studies showing a protective role for PDI in neurodegenerative diseases (Parakh et al. 2020, 2021; Walker et al. 2010), but a role against DNA damage has not been previously described. We demonstrate that PDI re‐locates to the nucleus following DNA damage, where it localises at γH2AX and 53BP1 foci, key proteins that facilitate DSB repair. There was also a trend towards co‐localisation of PDI with another DSB repair protein, DNA‐PK, but this did not reach statistical significance. Both treatments used in this study produce DSBs: etoposide inhibits topoisomerase II (Li et al. 2014), and H_2_O_2_ induces ROS, resulting in DSBs and single‐stranded breaks (SSB) (Sharma et al. 2016). Phosphorylation of H2AX is one of the earliest cellular responses to DSBs (Siddiqui et al. 2015) and during NHEJ, γH2AX recruits 53BP1 to DSB sites, where it promotes the ligation of distal DNA ends (Paull et al. 2000; Zhang et al. 2023). Treatment of cells with NU7741 confirmed that PDI mediates NHEJ.

In contrast, the PDI QUAD mutant was not protective against DNA damage, revealing that the two active site cysteines, and thus the redox activity of PDI, mediate the repair function. This finding identifies the specific region of PDI with protective activity against DNA damage, with implications for the design of future possible therapeutic interventions. The therapeutic potential of PDI in vivo was also highlighted by its protective activity against DNA damage in zebrafish, confirming that it is effective in a whole organism. The QUAD mutant was also not protective in zebrafish, confirming that the redox function is required for the repair function in vivo. We considered zebrafish a suitable model to validate our in vitro findings (Cayuela et al. 2018) because DSB and NHEJ (Takata et al. 1998) can be induced by H_2_O_2_ (Reinardy et al. 2013b), but become rapidly neutralised, with no residual perseverance in this organism (Chuang et al. 2002; Reeves et al. 2008).

There is increasing evidence that homeostasis of the proteome and genome are intimately associated (Shadfar et al. 2023). However, while other chaperones have been linked to the DDR, including cytoplasmic heat shock proteins 27, 70, and 90 (Knighton and Truman 2019; Mendez et al. 2000), here we show that the chaperone activity of PDI alone is not protective against DNA damage. The requirement of the redox function of PDI in DNA repair thus identifies a new link between cellular redox regulation and DNA damage, consistent with increasing evidence linking the two mechanisms (Shadfar et al. 2023). Some proteins function in both processes (Ainslie et al. 2021), such as apurinic/apyrimidinic endonuclease/redox factor‐1 (APE‐1), which mediates both DNA repair (Luo et al. 2010) and redox regulation of several transcription factors (Fishel et al. 2015; Feroz and Sheikh 2020). Similarly, p53 is a well‐characterised DDR protein and “guardian of the genome” (Feroz and Sheikh 2020) that also functions in redox homeostasis (Eriksson et al. 2019). Ku is part of the DNA‐PK complex that functions in NHEJ, but it also binds to DNA via redox activity (Andrews et al. 2006). Similarly, this study shows that PDI has dual functionality in redox regulation and DNA damage. It remains unclear how the redox activity of PDI functions in NHEJ, but there are several possibilities. Its localisation at γH2AX and 53BP1 foci implies that it may regulate the activity of these proteins. Alternatively, PDI may modulate the cellular redox status or activate redox‐regulated DDR transcription factors bearing zinc‐finger motifs or transition metal binding regions (Sun and Oberley 1996). PDI initiates the binding of transcription factors nuclear factor‐κB (NF‐κB) and AP‐1 to DNA (Clive and Greene 1996) and with p53, NF‐κB alters the transactivation of several DDR genes (Wang et al. 2017).

PDI is involved in the growth, metastasis, and survival of tumour cells and has been implicated as a target for chemotherapy (Powell and Foster 2021; Xu et al. 2019). Here we show that PDI prevents DNA damage induced by at least two mechanisms in two neuroblastoma cell lines. Consistent with our observations, knockdown of PDI in glioblastoma cells resulted in downregulation in transcription of DNA repair genes (Xu et al. 2019). Genes involved in multiple DNA repair pathways were identified in this previous study, including NHEJ, base excision repair, mismatch repair, and HR, implying that PDI functions in several DNA damage mechanisms (Terada et al. 1995). However, it was not previously shown that PDI is protective against DNA damage, nor determined whether PDI is directly or indirectly related to the DDR. In contrast, our findings reveal that PDI functions directly in the DDR, and that it is protective against DNA damage via NHEJ DNA repair, specifically via the redox activity. The previous authors also speculated that PDI inhibition downregulates DNA repair genes by reducing expression of E2F transcription factors (Terada et al. 1995). However, this has been attributed to the UPR rather than a direct effect in DNA repair because there is cross‐talk between DNA damage and ER stress (Schroder and Kaufman 2005; Szegezdi et al. 2006), although this is poorly defined (Zheng et al. 2018). ER stress sensitises cells to genomic damage (Dicks et al. 2015), inducing apoptosis through p53 activation (Mlynarczyk and Fahraeus 2014), and pharmacological activation of the UPR induces DNA repair genes (Liu et al. 2019). The findings of this study provide further insights into these mechanisms. Protein misfolding in the ER can lead to oxidative and ER stress, and thus DNA damage. Our finding that the redox activity of PDI specifically protects against DNA damage raises the possibility that its role in ensuring proper disulphide bond formation, and thus protein folding in the ER, may mediate its protective function in the DDR.

There are also other possible mechanisms implied by the findings of our study, although further studies are required to define these events. Cysteine residues in the CGHC active site of PDI can act as redox buffers by undergoing reversible oxidation and reduction (Hatahet and Ruddock 2009). This would allow PDI to scavenge excess ROS by forming disulphide bonds via its cysteine thiols. As ROS can induce DSBs that are repaired by NHEJ, this mechanism would therefore prevent them from damaging DNA. Similarly, the cysteine residues of PDI can also influence the activity of thiol‐dependent antioxidant systems, such as thioredoxin and glutathione peroxidases (Ulrich and Jakob 2019), by facilitating the regeneration of antioxidants, which could also neutralise ROS and thus prevent the induction of DNA damage. Some NHEJ proteins contain numerous cysteine residues, including DNA‐PKcs, Ku70/Ku80, XRCC4, and Ligase IV. It is also possible that the oxidative modification of these proteins via the active site cysteine residues of PDI could influence their functions. This may involve structural conformation of DNA‐PKcs, thereby affecting its kinase activity, or modulation of the DNA‐binding properties of Ku70/Ku80, thereby affecting the overall efficiency of NHEJ. Further studies into these possible mechanisms are therefore warranted.

PDI is primarily located in the ER, but it has been detected in multiple cellular locations (Terada et al. 1995), particularly on the cell surface (Terada et al. 1995; Shergalis and Neamati 2018; Turano et al. 2002). Following specific types of cellular stress, it translocates into the cytoplasm via a “reflux” mechanism (Igbaria et al. 2023). Here, we show that a proportion of PDI relocates into the nucleus following DNA damage, to discrete γH2AX and 53BP1 foci. Endogenous PDI was examined, implying that this may be a normal, but uncharacterised, physiological response. Consistent with our observations, several studies have detected PDI within the nucleus of various cell types (Turano et al. 2002; Coppari et al. 2002), where it anchors to the nuclear matrix via a redox‐dependent mechanism (VanderWaal et al. 2002).

## 5 Conclusion

**FIGURE 8:**
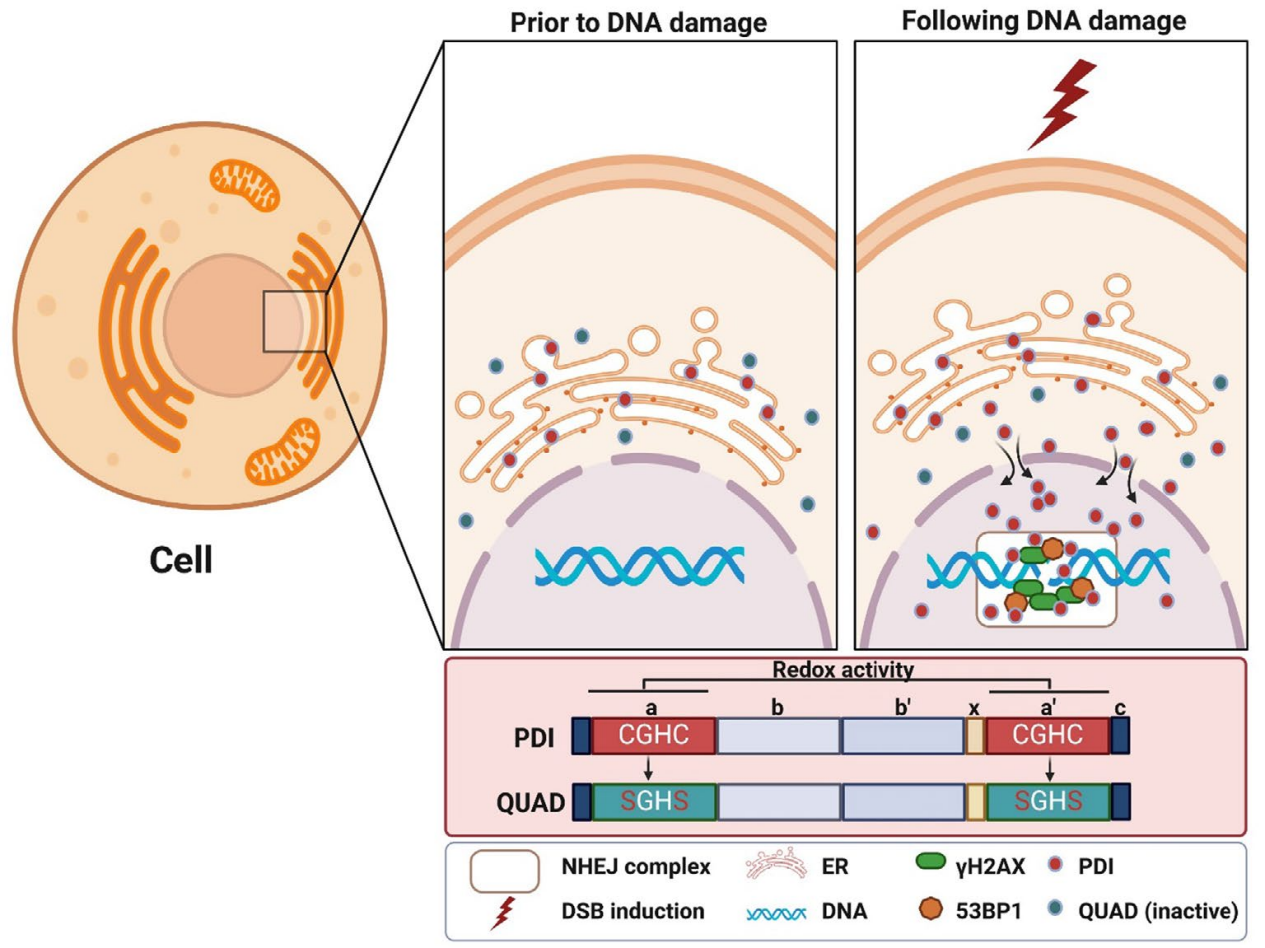
Schematic diagram illustrating the protective role of PDI against DSB DNA damage via NHEJ. Induction of DNA damage results in the formation of γH2AX and 53BP1 foci during NHEJ repair of DSBs. PDI (red circles) translocates into the nucleus following damage, where it co‐localises at DSB foci, facilitating NHEJ DNA repair. The PDI QUAD (green circles) contains mutations in which all four redox‐active cysteine residues are replaced with serine (SGHS SGHS). This mutant loses the redox activity but retains the chaperone function of PDI. It is not protective against DNA damage, demonstrating that the redox activity of PDI is required for its function in NHEJ. Figure created with BioRender.com.

There are currently no effective strategies to enhance DNA repair in ageing or neurodegenerative diseases, and inducing DNA damage is a therapeutic approach to selectively kill tumour cells. Here, we demonstrate that PDI plays a role in NHEJ repair of DSBs in neuroblastoma cells and mouse primary neurons, indicating that it has a protective function not only in neoplastic cells, but also in post‐mitotic neurons where NHEJ is the primary repair mechanism. The protective activity is localised to two cysteine residues within the two active sites, highlighting this region as a future therapeutic target. We also confirm the protective function of the redox activity of PDI in a whole organism. Hence, harnessing the redox function of PDI has potential as a therapeutic target in a range of human diseases (Figure 8).

## Author Contributions

S.S. (Shadfar) performed immunocytochemistry, Western blotting studies, and all other experiments except where indicated. F.F. performed PDI knockdown studies. S.S. (Saravanabavan) performed the Comet assay. C.J.J. performed immunocytochemistry analysis. A.M.R. and D.R.W. performed super‐resolution microscopy. S.P. and M.B. provided intellectual input. S.S., K.C.Y., and A.S.L. performed zebrafish studies. E.P. performed isolation and culturing of mouse primary cortical neurons. J.D.A. conceived and supervised the project, and provided intellectual input throughout. S.S. (Shadfar) and J.D.A. wrote the manuscript, with input from all co‐authors. All authors critically revised the intellectual content of the manuscript.

## Ethics Statement

All animal husbandry and experimental procedures were performed in compliance with the Animal Ethics Committee, Macquarie University (ARA 2015/034, ARA 2017/019, ARA 2017/03‐021) and the Internal Biosafety Committee, Macquarie University (NLRD 5201401007, 5974 52019597412350).

## Consent

The authors have nothing to report.

## Conflicts of Interest

The authors declare no conflicts of interest.

## Supporting information


**Figure S1.** PDI inhibits the formation of γH2AX and 53BP1 DNA damage foci induced by etoposide in NSC‐34 cells. (A, C) NSC‐34 cells overexpressing PDI tagged with V5 (red) or empty vector pcDNA3.1(+) were subjected to immunocytochemistry following treatment with 13.5 μM etoposide for 30 min at 24 h post‐transfection, using anti‐ γH2AX (A) or anti‐53BP1(C) (both green) and V5 antibodies (red). Nuclei were stained with Hoechst (blue). Arrows represent γH2AX foci (A) 53BP1 foci (C); Scale bar: 10 μm. One‐way ANOVA followed by Tukey’s multiple comparison post hoc test, ****p ≤ 0.0001, mean ± SEM. (B, D) Quantification of the average number of foci per 100 cells shown in (A, D); PDI over‐expression significantly reduced the formation of γH2AX and 53BP1 foci in NSC‐34 cells, following etoposide‐induced DNA damage, One‐way ANOVA followed by Tukey’s multiple comparison post hoc test, ****p ≤ 0.0001, mean ± SEM.
**Figure S2.** PDI translocates to the nucleus following PDI overexpression in Neuro‐2a cells. (A) Neuro‐2a cells overexpressing PDI (red) or empty vector pcDNA3.1(+) were subjected to immunocytochemistry following treatment with 13.5 μM etoposide (or DMSO) for 30 min at 24 h post‐transfection, using anti‐γH2AX (green) and V5 antibodies (red). Nuclei were stained with Hoechst (blue). Scale bar: 10 μm. Confocal microscopy revealed the presence of PDI in the nucleus following DNA damage. (B, C) Western blotting of nuclear (B) and cytoplasmic (C) fractions prepared from lysates of cells expressing empty vector (EV), PDI, or untransfected cells (UT). An anti‐PDI antibody was used to detect PDI, and antibodies against lamin B and GAPDH were used as markers of the nucleus and GAPDH, respectively. (D, E) Quantification of PDI levels in the nuclear and cytoplasmic fractions of the blots shown in (B, C), following etoposide or DMSO treatment. The graph depicts the relative band intensity by densitometry of PDI relative to Lamin B in the nuclear fractions, and of PDI to GAPDH in the cytoplasmic fractions. The levels of PDI in the nucleus increased significantly following etoposide treatment. *n* = 3, **p < 0.001, ns = non‐significant. One‐way ANOVA followed by Tukey’s multiple comparison post hoc test. All values represent mean ± SEM.
**Figure S3.** Optimisation of the induction of DNA damage using H_2_O_2_ in zebrafish embryos. (A) Western blot analyses using anti‐γH2AX and anti‐GAPDH antibodies in zebrafish embryo lysates following 24‐h treatment with different H_2_O_2_ concentrations. (B) Quantification of blots in (B) using densitometry, GAPDH was used as a loading control. The graph depicts the relative band density of γH2AX to GAPDH. A dose‐ dependent induction of DNA damage was evident following treatment with increasing doses of H_2_O_2_, *n* = 1.

## Data Availability

The data that support the findings of this study are available on request from the corresponding author.
