# Correction to ‘The Redox Activity of Protein Disulphide Isomerase Functions in Non‐Homologous End‐Joining Repair to Prevent DNA Damage’

**DOI:** 10.1111/acel.70337

**Published:** 2025-12-23

Shadfar, S., Farzana, F., Saravanabavan, S., et al. (2025), The Redox Activity of Protein Disulphide Isomerase Functions in Non‐Homologous End‐Joining Repair to Prevent DNA Damage. *Aging Cell*, 24: e70079. https://doi.org/10.1111/acel.70079.

In the published version of the above article, we would like to make the following corrections:

**1. Comet assay—Figure 3C was labelled incorrectly.**

The third column of Figure 3C was incorrectly labelled as ‘PDI + NU7441’; this should be corrected to ‘Etoposide + NU7441’.

**2. Comet assay—Figure 3D was labelled incorrectly.**

The x‐axis of this graph was incorrectly labelled

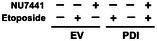

This should be corrected to

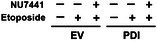

We apologize for these errors.

