# Supplementary material for: The Redox Activity of Protein Disulphide Isomerase Functions in Non‐Homologous End‐Joining Repair to Prevent DNA Damage
Source: Aging Cell. 2025 May 15;24(7):e70079. doi: 10.1111/acel.70079 (PMC12266753; doi:10.1111/acel.70079)
Supplement: Supplementary file 1 — Figure S1. PDI inhibits the formation of γH2AX and 53BP1 DNA damage foci induced by etoposide in NSC‐34 cells. (A, C) NSC‐34 cells overexpressing PDI tagged with V5 (red) or empty vector pcDNA3.1(+) were subjected to immunocytochemistry following treatment with 13.5 μM etoposide for 30 min at 24 h post‐transfection, using anti‐ γH2AX (A) or anti‐53BP1(C) (both green) and V5 antibodies (red). Nuclei were stained with Hoechst (blue). Arrows represent γH2AX foci (A) 53BP1 foci (C); Scale bar: 10 μm. One‐way ANOVA followed by Tukey’s multiple comparison post hoc test, ****p ≤ 0.0001, mean ± SEM. (B, D) Quantification of the average number of foci per 100 cells shown in (A, D); PDI over‐expression significantly reduced the formation of γH2AX and 53BP1 foci in NSC‐34 cells, following etoposide‐induced DNA damage, One‐way ANOVA followed by Tukey’s multiple comparison post hoc test, ****p ≤ 0.0001, mean ± SEM. Figure S2. PDI translocates to the nucleus following PDI overexpression in Neuro‐2a cells. (A) Neuro‐2a cells overexpressing PDI (red) or empty vector pcDNA3.1(+) were subjected to immunocytochemistry following treatment with 13.5 μM etoposide (or DMSO) for 30 min at 24 h post‐transfection, using anti‐γH2AX (green) and V5 antibodies (red). Nuclei were stained with Hoechst (blue). Scale bar: 10 μm. Confocal microscopy revealed the presence of PDI in the nucleus following DNA damage. (B, C) Western blotting of nuclear (B) and cytoplasmic (C) fractions prepared from lysates of cells expressing empty vector (EV), PDI, or untransfected cells (UT). An anti‐PDI antibody was used to detect PDI, and antibodies against lamin B and GAPDH were used as markers of the nucleus and GAPDH, respectively. (D, E) Quantification of PDI levels in the nuclear and cytoplasmic fractions of the blots shown in (B, C), following etoposide or DMSO treatment. The graph depicts the relative band intensity by densitometry of PDI relative to Lamin B in the nuclear fractions, and of PDI to GAPDH in th [file ACEL-24-e70079-s001.docx]

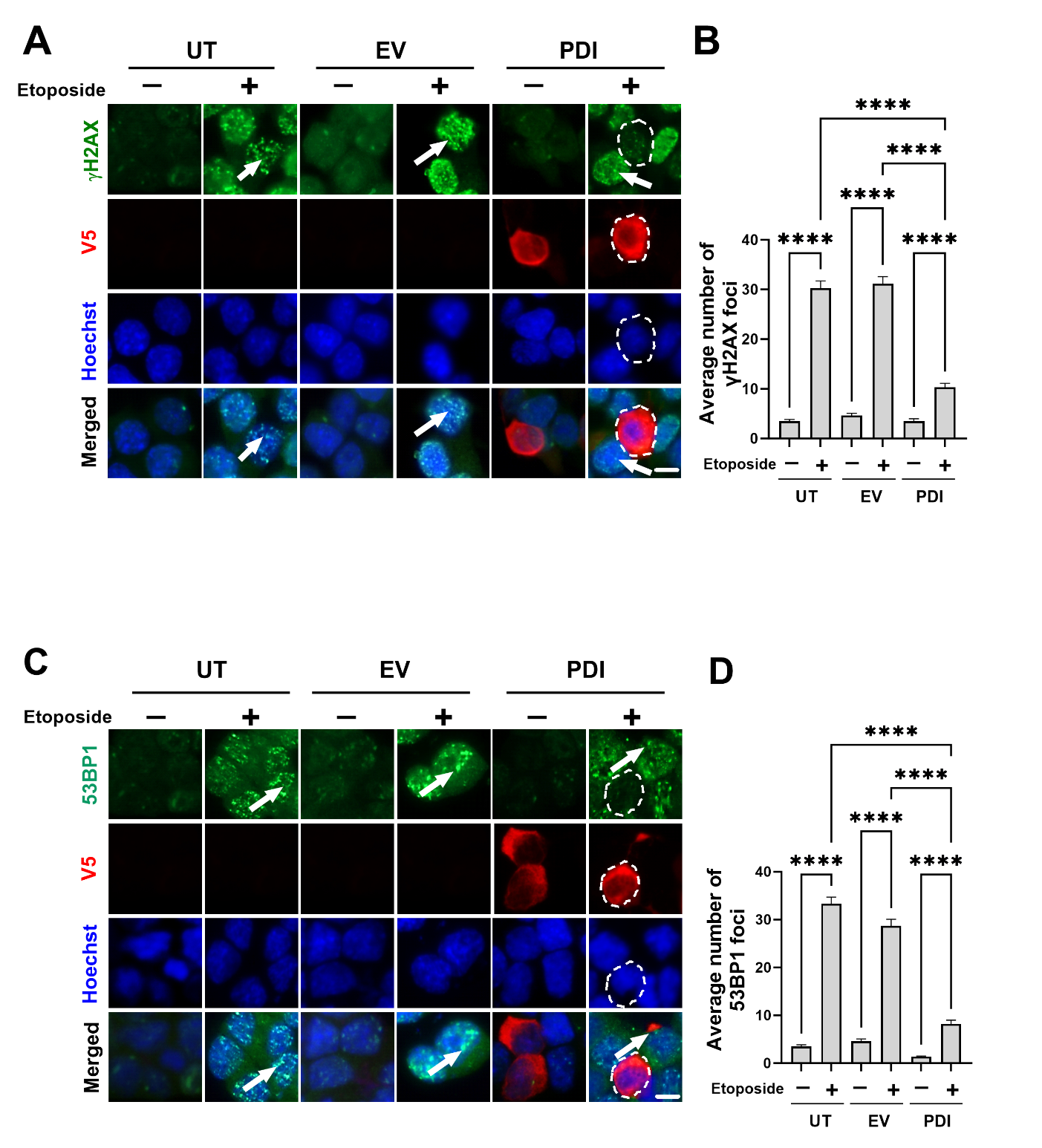
Supplementary Fig. 1 PDI inhibits the formation of γH2AX and 53BP1 DNA damage foci induced by etoposide in NSC-34 cells. A, C) NSC-34 cells overexpressing PDI tagged with V5 (red) or empty vector pcDNA3.1(+) were subjected to immunocytochemistry following treatment with 13.5 µM etoposide for 30 minutes at 24 hours post-transfection, using anti- γH2AX (A) or anti-53BP1(C)(both green) and V5 antibodies (red). Nuclei were stained with Hoechst (blue). Arrows represent γH2AX foci (A) 53BP1 foci(C); Scale bar: 10 μm. One-way ANOVA was performed followed by Tukey’s multiple comparison *post-hoc* test, ****p ≤0.0001, mean ± SEM.

B, D) Quantification of the average number of foci per 100 cells shown in (A, D); PDI over-expression significantly reduced the formation of γH2AX and 53BP1 foci in NSC-34 cells, following etoposide-induced DNA damage, One-way ANOVA was performed followed by Tukey’s multiple comparison *post-hoc* test ****p ≤0.0001, mean ± SEM.


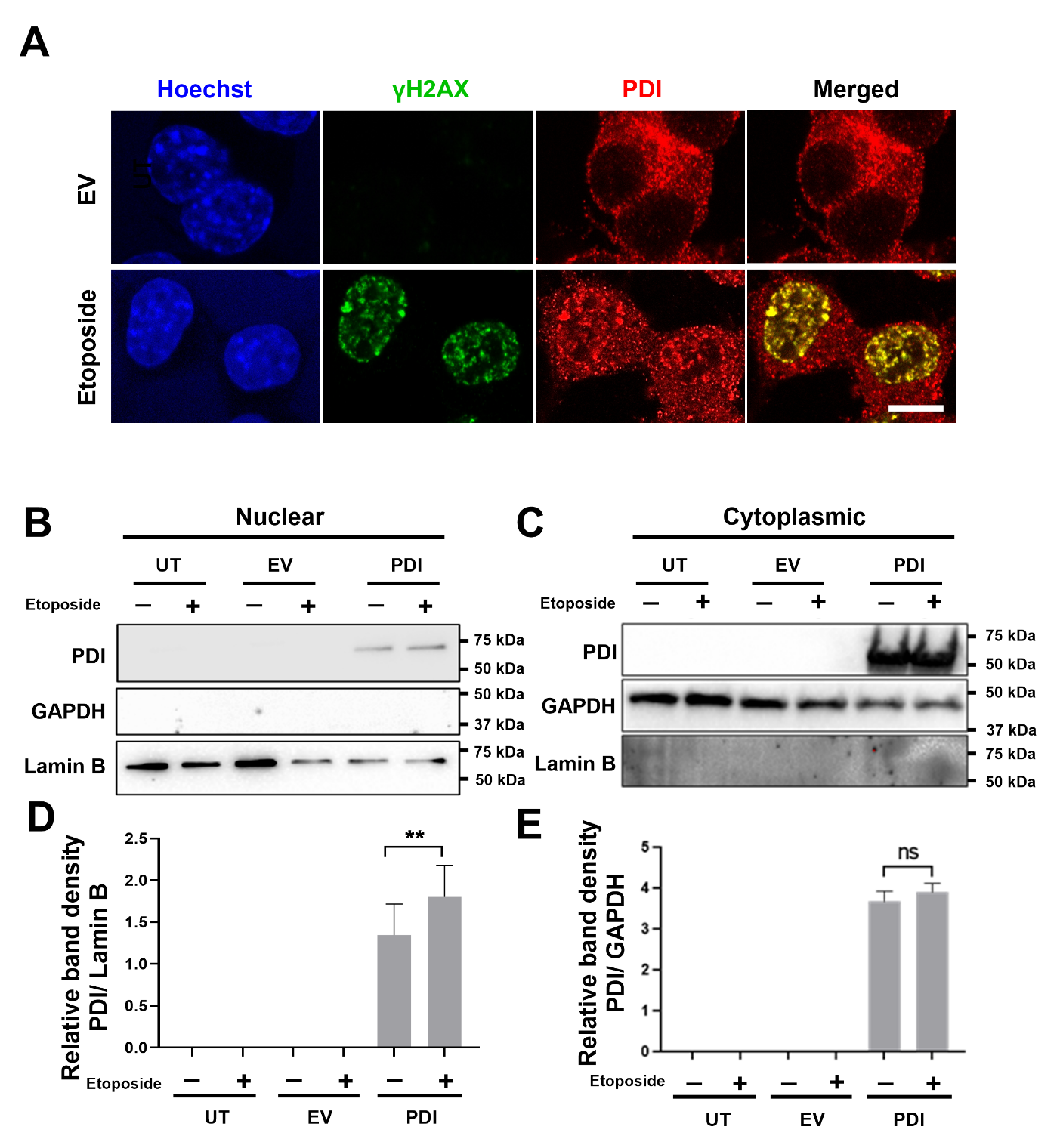


**Supplementary Fig. 2 PDI translocation into nucleus following PDI overexpression in Neuro-2A cells**

A) Neuro-2A cells overexpressing PDI (red) or empty vector pcDNA3.1(+) were subjected to immunocytochemistry following treatment with 13.5 µM etoposide for 30 minutes at 24 hours post-transfection, using anti- γH2AX (green) and V5 antibodies (red). Nuclei were stained with Hoechst (blue). Scale bar: 10 μm. Confocal microscopy revealed the presence of PDI in the nucleus following DNA damage B, C) Western blotting of nuclear (B) and cytoplasmic (C) fractions prepared from lysates of cells expressing empty vector or PDI, or untransfected cells. An anti-PDI antibody was used to detect PDI, and antibodies against lamin B and GAPDH were used as markers of the nucleus and GAPDH respectively. D, E) Quantification of PDI levels in the nuclear and cytoplasmic fractions of the blots shown in (B, C), following etoposide or DMSO treatment. The graph depicts the relative band intensity by densitometry of PDI relative to Lamin B in the nuclear fractions, and of PDI to GAPDH in the cytoplasmic fractions. The levels of PDI in the nucleus increased significantly following etoposide treatment. n=3, **p < 0.001, ns = non-significant. One-way ANOVA followed by Tukey's multiple comparison post-hoc test. All values represent mean ± SEM.


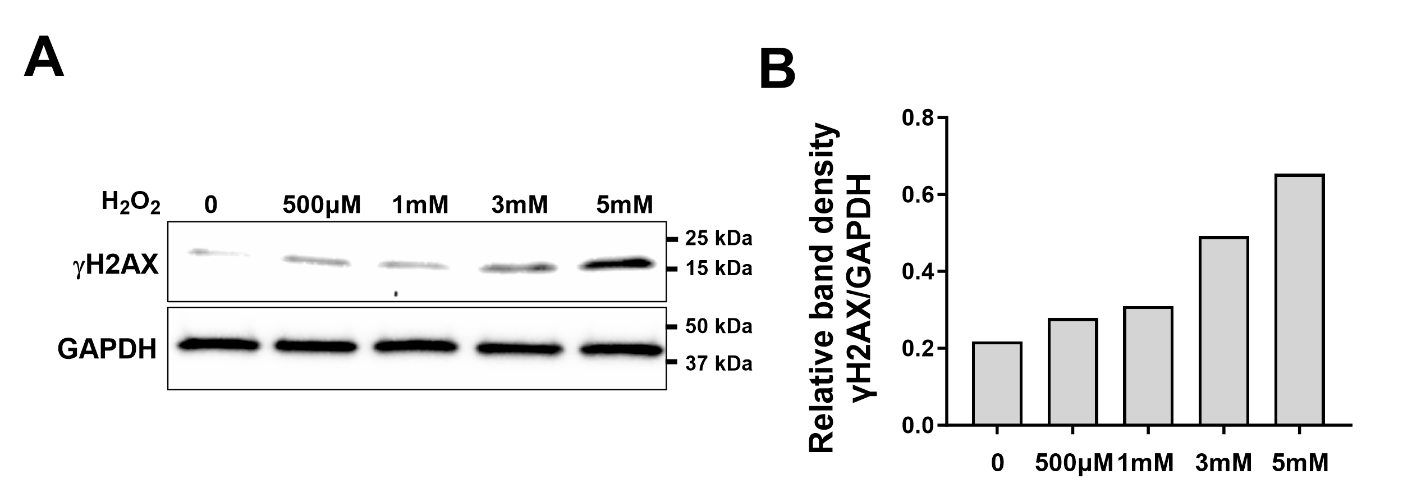


**Supplementary Fig. 3** **Optimization of the induction of DNA damage using different concentrations of H_2_O_2_ in zebrafish embryos** A) Western blot analyses using anti- γH2AX and anti-GAPDH antibodies in zebrafish embryo lysates following 24 hours treatment with different H_2_O_2_ concentrations. B) Quantification of blots in (B) using densitometry, GAPDH was used as a loading control. The graph depicts the relative band density of γH2AX to GAPDH showed the does dependant induction of double stranded DNA damage following treatment with different doses of H_2_O_2_. n=1
